# *Mytilus edulis*-mediated green synthesis of selenium nanoparticles with antimicrobial and molluscicidal applications

**DOI:** 10.1038/s41598-026-65860-3

**Published:** 2026-09-07

**Authors:** Hebat-Allah A. Dokmak, Moaz AlSedawy, Marwa A. Ramadan, Basma H. Amin, Amna H. Faid, Hagar F. Abdelmaksoud, Salwa M. El-Sayed

**Affiliations:** 1 Department of Medical Malacology, Theodor Bilharz Research Institute, Giza, Egypt; 2 Microbiology and Immunology Department, Faculty of Pharmacy (For Boys), Al-Azhar University, Cairo, Egypt; 3 Department of Laser Application in Metrology, Photochemistry and Agriculture, National Institute of Laser Enhanced Science (NILES) Cairo University (CU), Giza, Egypt; 4 The Regional Center for Mycology and Biotechnology, Al-Azhar University, Cairo, 11787 Egypt; 5 Department of Laser Science and Interaction, National Institute of Laser Enhanced Science (NILES) Cairo University, Giza, Egypt; 6 Department of Parasitology, Theodor Bilharz Research Institute, Giza, Egypt; 7 Department of Biochemistry, Faculty of Agriculture, Ain Shams University, Cairo, 11566 Egypt

**Keywords:** *Mytilus edulis*, Selenium nanoparticles, Green synthesis, GC–MS, Antimicrobial activity, *Biomphalaria alexandrina*, Genotoxicity, Biochemistry, Biological techniques, Biotechnology, Microbiology

## Abstract

This study investigated the bioactive profile of *Mytilus edulis* extract and its contribution to the green synthesis of selenium nanoparticles (ME-SeNPs), followed by comprehensive biological evaluation. HPLC analysis showed a high phenolic content dominated by gallic acid (61.25%) and chlorogenic acid (38.74%), indicating strong antioxidant potential. GC–MS profiling identified fifty compounds, mainly aromatic/heterocyclic constituents (37.37%) and lipid-derived constituents (37.04%), supporting diverse bioactivities and enabling nanoparticle biosynthesis through natural reducing and stabilizing agents. The successful formation of ME-SeNPs was confirmed by UV–Vis spectroscopy, evidenced by a surface plasmon resonance (SPR) peak at 260–350 nm and a clear visible color change. DLS measurements indicated moderate colloidal stability, with an average particle size of 292 nm and a zeta potential of − 33.13 mV. Morphological characterization by TEM and SEM revealed well-dispersed spherical nanoparticles with a size range of 35–65 nm. In addition, FTIR results verified the involvement of proteins and polysaccharides in the nanoparticle formation process. Biologically, ME-SeNPs exhibited significant antimicrobial activity. Testing against two standard bacterial strains demonstrated stronger inhibition of the Gram-negative bacterium *Escherichia coli* (MIC = 31.25 µg/mL) compared with the Gram-positive strain *Staphylococcus aureus* (MIC = 62.5 µg/mL). Regarding molluscicidal potential, the biogenic nanoparticles produced dose-dependent lethality against *Biomphalaria alexandrina*, with the median lethal concentration LC50 = 135.27 mg/L (95% CI 96.10–161.86 mg/L). The steep regression slope (1.18). Moreover, ME-SeNPs demonstrated dose-dependent molluscicidal effects in *Biomphalaria alexandrina*. However, sublethal exposure enhanced immune responses while concurrently inducing significant DNA damage.

**Supplementary Information:**

The online version contains supplementary material available at https://doi.org/10.1038/s41598-026-65860-3.

## Introduction

The blue mussel, *Mytilus edulis*, is a widely distributed marine bivalve belonging to the family *Mytilidae* and stands as one of the most extensively documented model organisms in marine biology^1–4^. Beyond its paramount role in global aquaculture and commercial fisheries, this species is recognized as a rich biogenic reservoir of bioactive macromolecules including high-quality proteins, functional peptides, polyunsaturated fatty acids (PUFAs), polyphenols, carotenoid pigments, essential minerals, and vitamins exerting potent antimicrobial, antioxidant, anti-inflammatory, antihypertensive, and cytoprotective properties^5,6^. Notably, specific protein hydrolysates derived from *M. edulis*, particularly the 50 kDa fraction, elicit robust antiproliferative effects, inducing high mortality rates against various malignant cell lines (including A549, BT549, HCT15, and PC3) even at markedly low concentrations^7^. Biogenic selenium nanoparticles (SeNPs) have attracted considerable biomedical interest due to strong antimicrobial performance paired with minimal cytotoxicity toward mammalian cells. Previous studies reported that SeNPs synthesized using microbial systems including *Staphylococcus aureus*, MRSA, *Escherichia coli*, and *Pseudomonas aeruginosa* effectively inhibit bacterial growth at concentrations of 25–250 µg/mL^8^. In addition, SeNPs biosynthesized by *Fusarium fujikuroi* MED14 have been shown to cause substantial structural degradation in multidrug-resistant strains such as *E. coli* MED10, *E. coli* ATCC 25922, and *Bacillus cereus* ATCC 14579^9^. Consequently, nanotechnology offers a transformative route for overcoming the limitations of conventional antibiotics in treating recalcitrant and intracellular bacterial infections. By optimizing cellular internalization, accelerating intracellular cargo accumulation, enabling controlled-release kinetics, and disrupting dense, resistant biofilms, nanoparticle-based delivery systems substantially enhance antimicrobial efficacy against persistent and multi-drug resistant pathogens^10,11^. In this regard, inorganic nanoparticles represent a promising alternative to conventional antibiotics by circumventing established microbial resistance mechanisms through distinct physical and biochemical pathways^12^.

Among candidate nanomaterials, SeNPs stand out for biomedical and environmental applications due to their broad-spectrum bioactivity, enhanced bioavailability, superior biocompatibility, and markedly lower toxicity compared with ionic selenium and metal-based counterparts^13,14^. The selection of *Biomphalaria alexandrina* as an experimental model is supported by its ecological and epidemiological role as the obligate intermediate host for schistosomiasis^15^. Moreover, freshwater snails serve as sensitive bioindicators for ecotoxicity evaluation: their high capacity to bioaccumulate contaminants drives severe histopathological damage, which is essential for defining environmental safety thresholds^15^. Importantly, a specific mature size class (8–10 mm shell diameter) was selected based on epidemiological criteria, because larger, mature specimens exhibit higher infection susceptibility and increased cercarial shedding rates thereby functioning as primary vectors that sustain transmission^21^. In parallel with these biomedical challenges, tropical public health crises persist, and schistosomiasis remains a major neglected tropical disease associated with severe socioeconomic burdens worldwide^15^,^16^. Because transmission depends strictly on specific freshwater snails acting as intermediate hosts particularly *Biomphalaria alexandrina* for *Schistosoma mansoni* vector eradication has long been established by the World Health Organization as a cornerstone of preventive strategies^17–20^. However, a critical paradigm shift is urgently needed regarding the selection of molluscicide agents. Although legacy chemical molluscicides, primarily niclosamide, are widely used for their immediate lethal action, their application is limited by significant drawbacks: niclosamide functions as a non-specific cellular toxin characterized by environmental persistence, high ecotoxicity, and acute risks to non-target aquatic organisms^20^. To address these ecological limitations, recent efforts have increasingly focused on biodegradable biogenic alternatives derived from botanical, microbial, and marine sources to achieve a sustainable balance between efficacy and biosafety. Unlike the non-selective chemical toxicity of niclosamide, botanical bioactive compounds (e.g., *Phytolacca dodecandra* and *Lantana camara*), essential oils, and cyanobacterial metabolites can induce targeted physiological disruption in snails while remaining environmentally benign^17,20,22^. Similarly, highly selective microbial toxins such as those from *Pseudomonas fluorescens* can effectively control invasive mollusk populations without causing mammalian toxicity^23–25^.

This eco-friendly advantage is further supported by phycocyanin extracts from *Anabaena oryzae*, *Nostoc muscorum*, and *Spirulina platensis*, which show high lethality against *B. alexandrina* while remaining harmless to non-target aquatic fauna^26^. Bridging natural product chemistry and nanotechnology, Lyu et al. reported that functionalized selenium nanoparticles display significant antimicrobial activity with negligible toxicity in zebrafish models^27^. The key novelty of this study lies in utilizing the native biochemical matrix of *Mytilus edulis* as an integrated dual-function system, where its endogenous biomolecules simultaneously reduce and stabilize selenium nanoparticles (ME-SeNPs) a process visually confirmed by a distinct red–orange color transition. This intrinsic biomolecular corona, dominated by gallic acid (61.25%) and chlorogenic acid (38.74%), bypasses conventional plant- or microbe-based synthesis routes. Functionally, this unique chemical signature combats antimicrobial resistance (AMR) by disrupting *Staphylococcus aureus* and *Escherichia coli* strains , while concurrently targeting the intermediate host, *Biomphalaria alexandrina*, through oxidative stress and pronounced genotoxicity, as evidenced by elevated DNA strand breaks in the comet assay.

## Material and methods

### Mussels (*Mytilus edulis*) sampling

Specimens of *Mytilus edulis* were collected from the coastal waters of Ain Sokhna, along the Egyptian Red Sea shoreline. Sampling was conducted using a systematic random sampling design to ensure that the collected specimens were representative of the local population, with individuals randomly selected from multiple intertidal sites to minimize spatial bias. The collected mussels were carefully transported in clean containers filled with ambient seawater to the Medical Malacology Laboratory, Theodor Bilharz Research Institute, Egypt, for further processing and analysis. Species identification was confirmed according to standard marine taxonomic keys and classification references of marine bivalves. licences, or authorizations for collecting wild mussels or confirmation of compliance with relevant regulations: Not Applicable.

### Preparation of the ethanolic extract of *Mytilus edulis*

*Mytilus edulis* specimens were carefully removed from their shells, and the soft tissues were thoroughly washed with tap water to eliminate adhering debris. The tissues were then minced and freeze-dried using a lyophilizer. A 5 g portion of the lyophilized material was extracted with 50 mL of 80% ethanol for 24 h at 4 °C. Following this, the mixture was centrifuged at 6000 rpm for 5 min to collect the supernatant. To ensure maximum recovery of bioactive constituents, the residual biomass was re-extracted under identical conditions for three consecutive cycles. The resulting supernatants were pooled and concentrated under reduced pressure at 40 °C using a rotary evaporator. Finally, the concentrated extract was freeze-dried and stored at 4 °C for subsequent phytochemical separation and quantitative determination via HPLC and GC–MS analyses, as previously described^29,30^.

### Separation and quantification of phenolic compounds by high-performance liquid chromatography (HPLC) of the lyophilized *Mytilus edulis* extract

For the determination of phenolic constituents, 0.2 g of the lyophilized *Mytilus edulis* extract was dissolved in 1 mL of HPLC-grade methanol. The mixture was centrifuged, and the resulting supernatant was used for HPLC analysis. Chromatographic separation was conducted using an Agilent 1260 series system equipped with a Zorbax Eclipse Plus C18 column (4.6 × 250 mm, 5 μm particle size), following procedures adapted from Gökbulut et al.^31^. The mobile phase consisted of water (A) and acetonitrile containing 0.05% trifluoroacetic acid (B), delivered at a flow rate of 0.9 mL/min. A linear gradient program was applied to optimize the separation: 60% A from 8 to 12 min, increasing to 82% A from 12 to 16 min, and maintained at 82% A until 20 min. The injection volume was 5 μL, and the column temperature was held at 40 °C. Peak identification and quantification were performed based on retention times compared to authentic standards.

### Separation and identification of bioactive compounds by gas chromatography–mass spectrometry (GC–MS) in dried extract of *Mytilus edulis*

The dried extract of *Mytilus edulis* was reconstituted in 3 mL of ethyl acetate, and 1 mL of the solution was transferred to a GC vial for analysis. Gas chromatography–mass spectrometry (GC–MS) was employed to profile both the major and minor components of the ethanolic extract. Component identification was achieved by comparing their mass spectra and retention times with those of authentic standards, as well as by computer-assisted matching against the NIST and WILEY mass spectral libraries. Fragmentation patterns were further cross-checked with published literature for confirmation^30^. The analysis was performed using an Agilent 7890A GC system coupled with an Agilent 7000 mass-selective detector (MSD) and equipped with a polar HP-5ms capillary column (5% phenyl methyl polysiloxane, 30 m × 0.25 mm i.d., 0.25 μm film thickness). Helium was used as the carrier gas at a linear velocity of 1 mL/min. The injector and detector temperatures were set at 200 °C and 250 °C, respectively, with a sample injection volume of 1 μL. The MS was operated under the following conditions: electron ionization at 70 eV, interface temperature 250 °C, and mass acquisition range of 50–800 m/z.

## Selenium source

Selenium nanoparticles (SeNPs) were synthesized/obtained using sodium selenite (Na_2_SeO_3_) as a selenium precursor, purchased from Sigma-Aldrich (St. Louis, MO, USA).

### Biological synthesis of selenium nanoparticles (ME-SeNPs) using *Mytilus edulis* extract

**Fig. 1:**
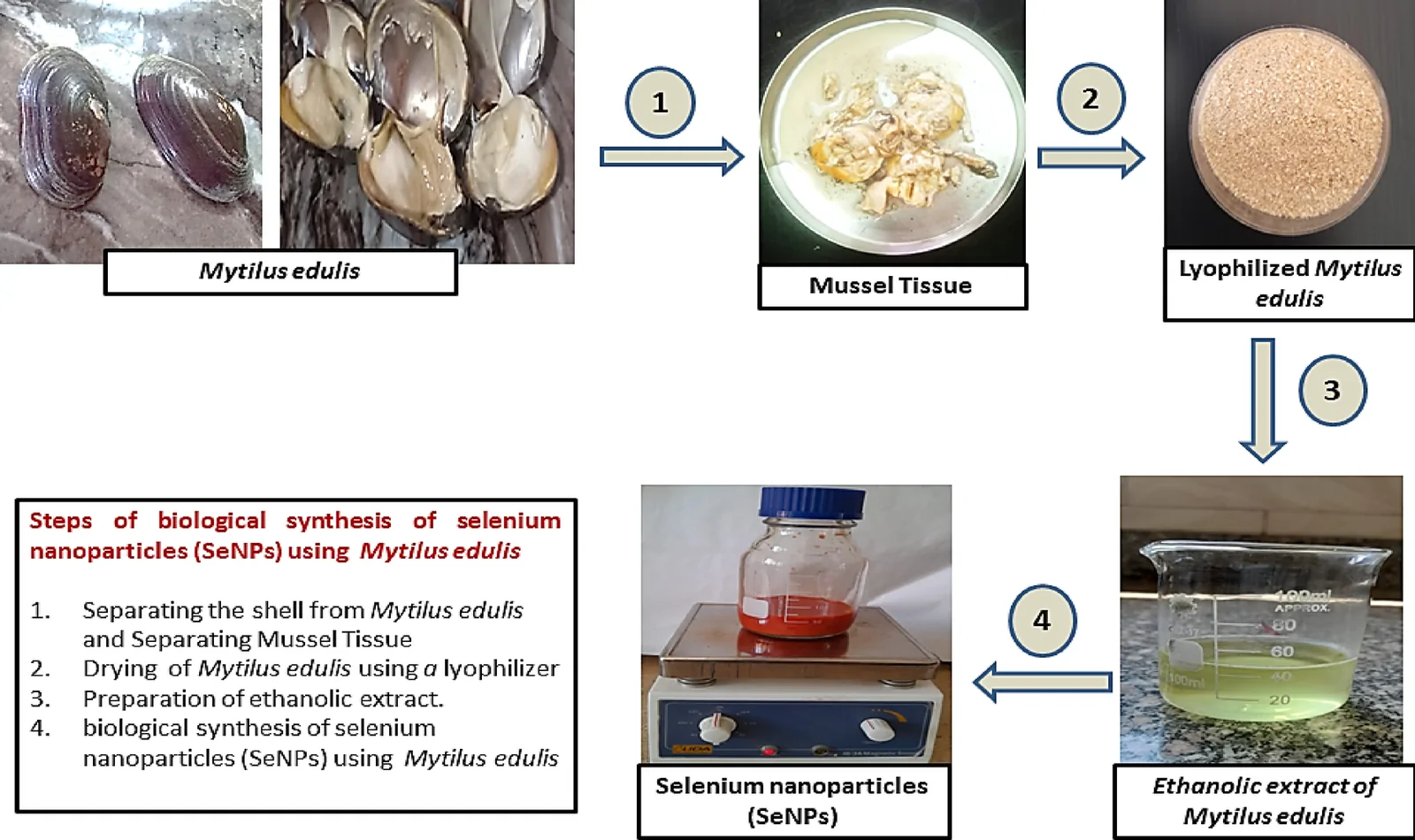
Biological synthesis of selenium nanoparticles (SeNPs) using ethanolic extract of *Mytilus edulis*.

Selenium nanoparticles (SeNPs) were biosynthesized by reducing selenium ions from sodium selenite (Na_2_ SeO_3_) using the ethanolic extract of *Mytilus edulis* as a natural reducing agent. Briefly, 10 mL of the fresh mussel extract was mixed with 90 mL of a 0.2 mM (Na_2_ SeO_3_) solution. The reaction mixture was homogenized on a magnetic stirrer for 2 h at room temperature (pH 6.8). A visible color change from yellow to red was observed, indicating the reduction of selenite ions and the successful formation of SeNPs. The reaction progress was monitored under continuous stirring, with observations recorded at 24-h intervals (Fig. 1). Following the completion of the color transformation, the synthesized SeNPs were collected and purified by washing several times with deionized water and centrifuging at 6000 rpm for 20 min, as previously described^32^. This approach demonstrates a simple, eco-friendly method for producing SeNPs using bioactive compounds present in *M. edulis*, providing a solid foundation for further physicochemical characterization and biological evaluation.

## Characterization of selenium nanoparticles (ME-SeNPs)

The optical properties of the synthesized samples were analyzed using UV–Visible spectroscopy. For this purpose, the sample was dissolved in deionized distilled water (DD water), and measurements were carried out using a 3 mm quartz cuvette. The absorption spectra were recorded using a double-beam UV–Vis–NIR spectrophotometer (Cary 5000, Agilent Technologies, Santa Clara, CA, USA)^33^.

The particle size distribution and surface charge (zeta potential) were evaluated using Dynamic Light Scattering (DLS). Measurements were performed using a Zetasizer 300 HAS (Malvern Instruments, Malvern, UK) based on photon correlation spectroscopy. The analysis time was set to 60 s, and the average particle size and zeta potential were determined without dilution of the nanoparticle dispersion^34^.

For morphological analysis, Transmission Electron Microscopy (TEM) was performed. A drop of the nanoparticle suspension was placed on carbon-coated copper grids (CCG) and allowed to dry at room temperature. The electron micrographs were obtained using a JEOL JEM-1010 transmission electron microscope operated at 80 kV at the Regional Center for Mycology and Biotechnology (RCMB), Al-Azhar University^35^.

Scanning Electron Microscopy (SEM) was used to investigate the surface morphology of the prepared BA-SeNPs. Samples were mounted on aluminum stubs using carbon adhesive tape and subsequently coated with a thin layer of gold (Au) for 120 s using a Quorum Technologies sputter coater (Q150T, England). Imaging was performed using a Tescan SEM system (TESCAN VEGA 3, Czech Republic)^36^,^37^.

Fourier Transform Infrared (FTIR) spectroscopy was employed to identify the functional groups involved in nanoparticle synthesis. Spectra were recorded using a Shimadzu FT-IR 8400 spectrometer in the range of 500–4500 cm^−1^^39^. For FTIR analysis, 4–8 mg of dried samples (ME and ME-SeNPs) were thoroughly ground with 200 mg of KBr to form pellets, ensuring minimization of scattering errors and accurate spectral readings^15,39–42^.

## Screening of antimicrobial activity of selenium nanoparticles (ME-SeNPs)

The antimicrobial activity of 500 µg/mL ME-SeNPs against standard stains of *S. aureus* ATCC 25923 and *E. coli* ATCC 25922 as examples for Gram-positive and Gram-negative bacteria respectively were assessed by using the well diffusion method on Muller-Hinton agar. 200 µg/mL of cefotaxime was used as a reference antibiotic for comparison. The plates were incubated for 24 h at 37 °C and the diameter of the inhibition zones produced by the ME-SeNPs and cefotaxime were measured in mm^43^.

### Determination of the minimum inhibitory concentration of selenium nanoparticles (ME-SeNPs) against *S. aureus *ATCC 25923 and *E. coli* ATCC 25922 strains

The minimum inhibitory concentrations (MICs) of ME-SeNPs against standard strains of *Staphylococcus aureus* (ATCC 25923) and *Escherichia coli* (ATCC 25922) were determined via a microdilution assay in 96-well plates using a two-fold serial dilution in triplicate. The ME-SeNPs were tested at an initial concentration of 500 µg/mL. The bacterial strains were cultured in Mueller–Hinton broth (Oxoid, UK) until reaching the exponential growth phase (2 × 10 ^8^ CFU/mL). This broth culture was subsequently diluted to achieve a final inoculum density of 5 × 10^5^ CFU/mL upon inoculation into each well containing the serial dilutions of the test sample. Following inoculation, the plates were incubated for 24 h at 37 °C. Cefotaxime (200 µg/mL) served as the reference standard for comparison. For a rigorous and standardized comparative evaluation, all concentrations for both agents were prepared and evaluated on an equivalent mass concentration basis (µg/mL), rather than molarity, owing to the polydisperse nature and complex biomolecular capping of the biogenic nanoparticles^44,45^.

### Time kill assay

For the time-kill assay, *S. aureus* (ATCC 25923) and *E. coli* (ATCC 25922) cultures in the logarithmic growth phase (OD_600_ > 0.800) were diluted to approximately ~ 10^6^ CFU/mL and exposed to ME-SeNPs at concentrations equivalent to × 0.5, × 1.0 and × 2.0 MIC their respective MICs. Aliquots were collected at designated time intervals (0, 2, 4, 6, 8, and 24 h) during incubation at 37 °C, and subsequently serially diluted in phosphate-buffered saline (PBS). The dilutions were plated onto trypticase soy agar (Oxoid, UK) and incubated at 37 °C for 18–20 h to determine viable colony counts^46^. Bactericidal activity was defined as a ≤ 3 log_10_ reduction (≤ 99.9\% killing) in CFU/mL relative to the initial inoculum, whereas bacteriostatic activity was defined as a stable bacterial population over time without significant CFU reduction^47,48^.

## Snail collection and maintenance

*Biomphalaria alexandrina* snails were obtained from the Medical Malacology Department, Theodor Bilharz Institute, Egypt. Only adult specimens with a shell diameter of 8–10 mm were used in the study. The snails were maintained individually in 10 plastic aquaria containing dechlorinated freshwater at 24–25 °C under a 12-h light/12-h dark photoperiod. They were fed ad libitum with dried leaf lettuce. The water in each aquarium was renewed weekly, and any deceased snails were promptly removed to maintain optimal experimental conditions.

### Snail groups

Freshwater *Biomphalaria alexandrina* snails (shell diameter 6–8 mm) were randomly assigned to six experimental groups, each containing ten snails, with three independent replicates per treatment to ensure statistical reliability.

- **Group I (Negative control):** Snails were maintained in dechlorinated water under the same experimental conditions without exposure to the nanomaterial.
- **Group II:** Exposed to 100 mg/L of ME-SeNPs for 24 h.
- **Group III:** Exposed to 125 mg/L of ME-SeNPs for 24 h.
- **Group IV:** Exposed to 150 mg/L of ME-SeNPs for 24 h.
- **Group V:** Exposed to 200 mg/L of ME-SeNPs for 24 h.
- **Group VI:** Exposed to 250 mg/L of ME-SeNPs for 24 h.

After the exposure period, all snails were carefully rinsed to remove residual nanomaterial and transferred to 1 L of aerated dechlorinated water maintained at 25 ± 1 °C for a 24-h recovery period before final observations.

### Exposure bioassay

A primary stock solution of the tested synthesized selenium nanoparticles (ME-SeNPs) was prepared at 1000 ppm on a weight-to-volume (w/v) basis. Serial dilutions were then made from the stock solution to evaluate the toxic effects of the synthesized selenium nanoparticles (ME-SeNPs) on *B. alexandrina* snails. Lethal concentration values (LC₅₀ and LC₉₀) were determined following exposure to the prepared concentration range using the statistical method described by Litchfield and Wilcoxon^49^.

### Assessment of selenium nanoparticles (ME-SeNPs) toxicity on *Biomphalaria alexandrina* snails

Experimental groups of *B. alexandrina* were established, with each group consisting of ten snails (shell diameter 6–8 mm) and three independent replicates per treatment to ensure statistical reliability. Snails were exposed to graded concentrations of ME-SeNPs (100, 125, 150, 200, and 250 mg/L) for 24 h. Following exposure, all snails were thoroughly rinsed to remove residual (ME-SeNPs) and transferred to 1 L of aerated dechlorinated water maintained at 25 ± 1 °C for a 24-h recovery period. A negative control group was maintained under identical environmental conditions without exposure to the nanomaterial. Mortality rates were recorded for each treatment and expressed as the mean of three replicates according to Nolan et al.^50^ The median lethal concentration (LC₅₀), 90% lethal concentration (LC₉₀), and the slope of the dose–response regression line was calculated using probit analysis, as described by Finny (1971), via Proban software^51^.

## Hemocyte proliferation

Hemoyte collected from adult *Biomphalaria alexandrina* snails.

Hemocyte was collected from adult *Biomphalaria alexandrina* snails by cardiac puncture. After disinfecting the shell with 70% ethanol, a sterile 1 mL syringe fitted with a 22-gauge needle was inserted near the heart to withdraw hemocyte directly into precooled sterile microtubes on ice for downstream analyses^52^.

### Hemocyte proliferation assay

Hemcyte proliferative activity was evaluated via the MTT reduction assay, reflecting mitochondrial metabolic function, following the method described by Rai-Elbalhaa et al.^53^. Briefly, heparinized blood samples were diluted 1:1 with Hank’s Balanced Salt Solution (HBSS), carefully layered over Ficoll-Hypaque at a 2:1 ratio, and centrifuged at 400 × g for 30 min at 4 °C. The hymocyte fraction was harvested from the interphase and washed twice with serum-free RPMI-1640 medium. Cell viability and density were determined using a hemocytometer, and the concentration was adjusted to 1 × 10^6^ cells/mL according to the following formula:

$$ {\mathrm{TotalHemocyte}} = {\text{ CountedHemocyte}} \times { 25 } \times { 1}0,000 \, \times {\text{ Dilution Factor}} $$

For the proliferation assay, the isolated Hemocyte were seeded into 96-well tissue culture plates in RPMI-1640 medium supplemented with 10% fetal calf serum. Mitogenic stimulation was induced using phytohaemagglutinin (PHA, Sigma) at a final concentration of 15 µg/mL. The cultures were incubated at 37 °C in a humidified 5% CO_2_ atmosphere for 72 h. Subsequently, MTT solution (5 mg/L in PBS) was added to each well, and the plates were incubated for an additional 5 h to facilitate the formation of blue formazan crystals. The resulting crystals were solubilized using 10% SDS in 0.01 M HCl over a 16-h incubation period. Absorbance was measured at 590 nm using a multiwell plate reader. After subtracting background values, the net increase in optical density was expressed as MTT units, serving as a direct indicator of hemocyte proliferation. To ensure experimental uniformity, this procedure was performed simultaneously under identical conditions for both the green selenium nanoparticles (ME-SeNPs)-treated group and the untreated control group snails.

## Nitric oxide determination

Snails of *Biomphalaria alexandrina* from both the control group and those exposed to sublethal concentrations (LC₁₀ and LC₂₅ ppm) of biosynthesized selenium nanoparticles were dissected, and the soft tissues were carefully separated from the shells. Tissue samples were pooled and homogenized at a ratio of 1 g tissue per 2 mL of dechlorinated water using an ultrasonic homogenizer (UP200H). The resulting homogenates were centrifuged at 4000 rpm for 45 min at 25 ± 1 °C. After removal of the cellular debris, the clarified supernatants were re-homogenized and subjected to an additional centrifugation step. Nitric oxide concentration in the final supernatants was determined using a commercial colorimetric assay kit (Biodiagnostic Co., Dokki, Giza, Egypt; Cat. No. GR 2511) according to the manufacturer’s instructions and previously established according to Montgomery and Dymock^54^.

## Assessment of DNA integrity using the comet assay

DNA damage was assessed using the comet assay in *Biomphalaria alexandrina* snails treated with sublethal concentrations (LC₁₀ and LC₂₅) of biosynthesized selenium nanoparticles. Results were compared with those obtained from untreated control snails. The assay was conducted following established protocols with minor modifications, as previously described Grazeffe et al.^55^.

### Statistical analyses

To ensure reproducibility, all antimicrobial efficacy evaluations encompassing zones of inhibition, Minimum Inhibitory Concentrations (MIC), and time-kill kinetics were conducted in triplicate (n = 3). Quantitative measurements for MIC values and inhibition zone diameters were expressed as mean ± standard deviation (SD) alongside 95% confidence intervals. For time-kill kinetics assays, statistical variations in log_10_ CFU/mL reductions across various time intervals were evaluated against the control group and initial inoculum using a two-way analysis of variance (ANOVA), followed by Dunnett’s post-hoc multiple comparisons test via GraphPad Prism software (Version 8).^56,57^ Statistical evaluations for *Biomphalaria alexandrina* snails were executed using GraphPad Prism and SPSS for Windows on an IBM-compatible PC to determine the significance of differences between the treated and control cohorts.^58^ Biochemical datasets were quantified as mean ± standard deviation (SD), and Student’s t-test was applied to assess the significance of differences between group means.^59^ Statistical significance was established at a 95% confidence level, with exact *p* values reported where applicable; thresholds of *p* < 0.05 and *p* < 0.001 were utilized to denote statistically significant and highly significant differences, respectively. Furthermore, mortality rates across the experimental and control groups were statistically validated and compared using Pearson’s Chi-square test^60^.

## Results

### Gas chromatography–mass spectrometry (GC–MS) based quantitative profiling of bioactive compounds in the ethanolic extract of *Mytilus edulis*

**Fig. 2:**
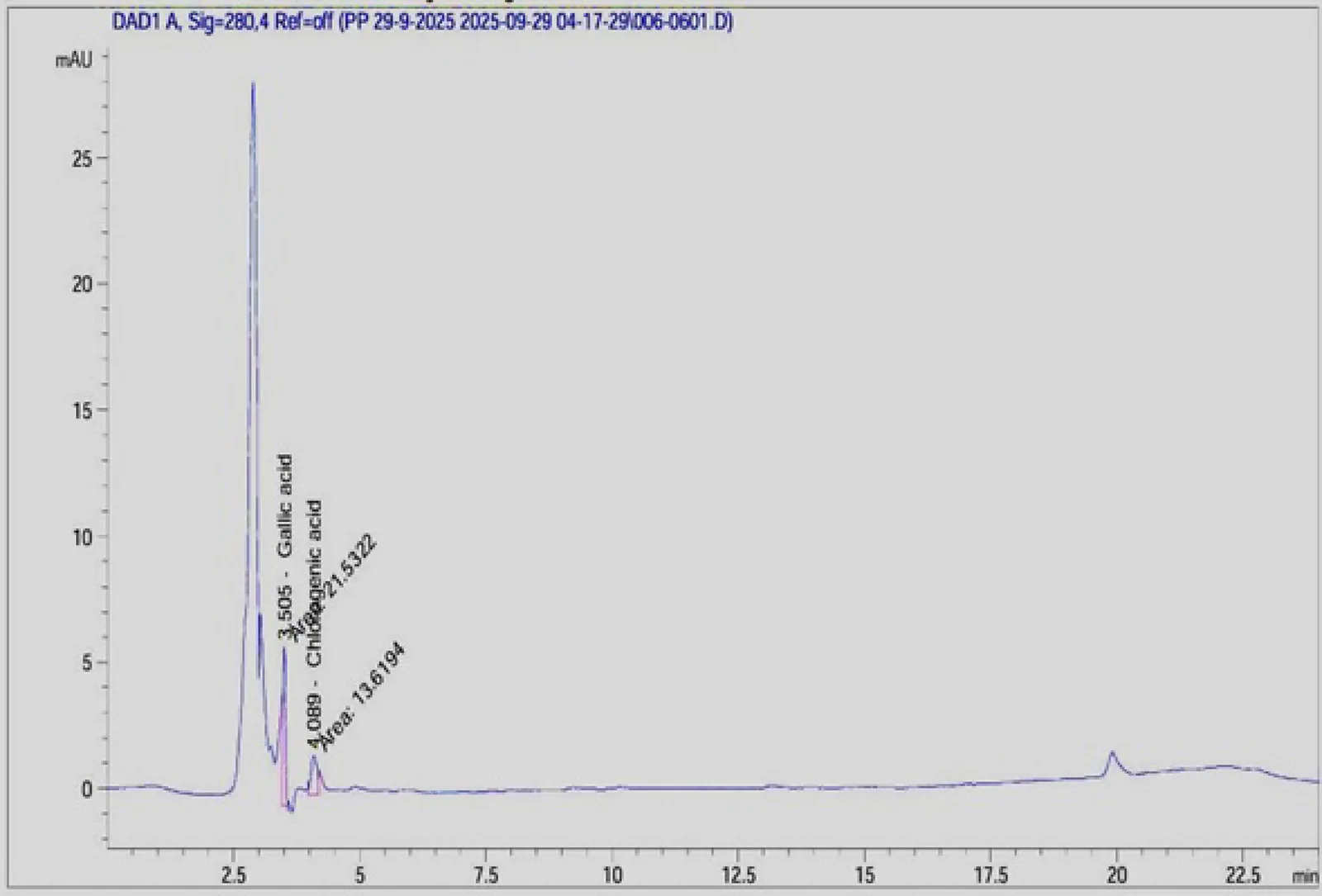
High-performance liquid chromatography (HPLC) chromatograms illustrating the profile of phenolic compounds identified in *Mytilus edulis*.

As presented in Table 1 and Fig. 2, HPLC analysis at 280 nm revealed that gallic acid (61.25%) and chlorogenic acid (38.74%) are the predominant phenolic acids in the *Mytilus edulis* extract. The presence of these major compounds supports the extract’s antioxidant capacity and its potential as a source of bioactive agents.

**Table 1:** Identification and quantification of phenolic compounds in *Mytilus edulis* using high-performance liquid chromatography (HPLC).

Phenolic Compounds in *Mytilus edulis*	RT (min)	Area [mAU*s]	%Area
Gallic acid	3.505	21.53	61.2553
Chlorogenic acid	4.089	13.62	38.7447

RT, Retention time; mAU*s, Milli-absorbance units times seconds (peak area units). Data represents the identification and quantification of major phenolic compounds in *Mytilus edulis* extracts. The percentage area (%Area) indicates the relative abundance of each compound based on total integrated peak area.

### Gas chromatography–mass spectrometry (GC–MS) based quantitative profiling of bioactive compounds in the ethanolic extract of *Mytilus edulis*

GC–MS analysis (Table 2) categorized the identified volatile and semi-volatile metabolites of the *Mytilus edulis* extract into seven major chemical families. Carbohydrates and sugar derivatives formed a structurally diverse fraction, dominated by D-arabinose (3.69%) and β-lyxopyranose (3.14%). Lipids and fatty acids represented a significant quantitative proportion, mainly driven by (Z)-9-hexadecenoic acid (18.54%) and 9-cycloheptadecen-1-ol (16.00%), reflecting a notable content of monounsaturated long-chain fatty acids and related alcohols. Alkaloids and nitrogenous heterocycles were also detected, with carbostyril derivatives and isobalfourodine reaching up to 6.47%. Other identified families included terpenoids (e.g., cholesterol), pyrans, lactones, and functionalized hydrazides. The presence of trimethylsilyl (TMS) and tert-butyldimethylsilyl (TBDMS) derivatives confirms successful derivatization, which enhanced the volatility and thermal stability of polar compounds for chromatographic separation. The detection of palmitic acid, palmitoleic acid derivatives, EPA, cholesterol, and related lipids aligns with previously reported lipid profiles of *M. edulis* (62, 63). Additionally, the identified carbohydrate and glycoside derivatives including levoglucosan, D-ribose, D-arabinose, lyxopyranose, lactose/lactulose/mannobiose derivatives, turanose, and N-acetylglucosamine are consistent with the established glycogen and glycoprotein/glycolipid composition of blue mussels (64–66).

**Table 2:** Gas chromatography–mass spectrometry (GC–MS)-based quantitative analysis of bioactive constituents in the Ethanolic Extract of *Mytilus edulis.*

Peak no	Compound name	RT [min]	%Area
1. *Fatty acids and derivatives*			
12	9-Hexadecenoic acid, (Z)-, TMS derivative	12.0638	18.5407
14	Pentadecanoic acid, TMS derivative	12.4701	0.0969
15	Palmitic Acid, TMS derivative	12.5330	0.0642
19	Eicosapentaenoic Acid, TBDMS derivative	13.6603	2.0868
21	Propanoic acid, 3-(trimethylsilyl)-, ethyl ester	13.9807	0.1078
39	Hexanedioic acid, 2TMS derivative	15.4055	0.0370
2. *Carbohydrates and sugar derivatives*			
2	Levoglucosan, 3TMS derivative	10.7707	0.0549
4	.beta.-D-Galactofuranoside, ethyl 2,3,5,6-tetrakis-O-(trimethylsilyl)-	10.9423	0.1341
5	.beta.-Lyxopyranose, 4TMS derivative	11.0797	3.1356
7	.alpha.-L-Mannofuranose, 6-deoxy-1,2,3,5-tetrakis-O-(trimethylsilyl)-	11.3371	2.3348
9	.alpha.-D-Galactopyranose, 1,2,3-tris-O-(trimethylsilyl)-, cyclic methylboronate	11.4687	2.4578
18	D-Lactose, octakis(trimethylsilyl) ether, methyloxime (isomer 2)	13.1739	0.2326
20	Butanal, 2,3,4-tris[(trimethylsilyl)oxy]-, O-methyloxime, [R-(R*,R*)]-	13.8662	0.4467
24	Lactulose, octakis(trimethylsilyl) ether, methyloxime (isomer 1)	14.2382	0.7357
26	2-.alpha.-Mannobiose, octakis(trimethylsilyl) ether (isomer 1)	14.3240	0.4232
27	2-.alpha.-Mannobiose, octakis(trimethylsilyl) ether (isomer 1)	14.4041	0.2697
30	D-Ribopyranose, 4TMS derivative	14.5929	2.4211
31	D-Arabinose, 4TMS derivative	14.6158	3.6875
36	D-Lactose, octakis(trimethylsilyl) ether, methyloxime (isomer 2)	14.9992	2.6099
37	D-Ribose, 4TMS derivative	15.1480	0.1091
42	N-Acetyl glucosamine methoxime, tetrakis(trimethylsilyl)	16.9504	0.0374
43	D-Ribose, 4TMS derivative	17.1278	0.0205
44	Methyl .alpha.-D-glucofuranoside, 4TMS derivative	17.2537	0.0940
45	threo-2,5-Hexodiulose, 1,3,4,6-tetrakis-O-(trimethylsilyl)-	17.4596	0.5159
46	Maltose, 8TMS derivative , isomer 1	17.6370	0.0885
47	.alpha.-D-Glucopyranose, 5TMS derivative	17.9575	0.0586
48	3-.alpha.-Mannobiose, octakis(trimethylsilyl) ether (isomer 2)	18.0147	0.0904
49	3-.alpha.-Mannobiose, octakis(trimethylsilyl) ether (isomer 1)	18.0948	0.0687
50	D-( +)-Turanose, octakis(trimethylsilyl) ether, methyloxime (isomer 1)	18.2664	1.3268
3. *Alkaloids and Nitrogen Heterocycles*			
6	Cyclopentene-1-carbonitrile, 2-(4-chlorophenyl)-	11.1483	5.4334
28	Carbostyril, 3-ethyl-4-(isopentyloxy)-7-methoxy-	14.4556	3.3983
32	Isobalfourodine	14.6959	5.0071
33	Carbostyril, 3-ethyl-4-(isopentyloxy)-6-methoxy-	14.8047	6.4660
34	Azepino[1,2-a]benzothieno[2,3-d]pyrimidin-13(1H)-one, 2,3,4,7,8,9,10,11-octahydro-4-hydroxy-	14.8848	3.8527
*4. Sterols and Terpenoids*			
3	1,2,4,8-Tetramethylbicyclo[6.3.0]undeca-2,4-diene	10.8794	1.4899
8	1,2,4,8-Tetramethylbicyclo[6.3.0]undeca-2,4-diene	11.4058	1.0374
41	Cholesterol, TMS derivative	16.7158	0.2390
*5. Pyrans, Lactones and Carbonyls*			
22	3,4-Dihydroxy-5-methyl-dihydrofuran-2-one, (D)-, 2TMS derivative	14.0894	0.0727
29	2H,6H-Benzo[1,2-b:5,4-b’]dipyran-6-one, 3,4-dihydro-5-hydroxy-2,2,8-trimethyl-	14.5243	3.5894
35	3,4-Heptadien-2-one, 3,5-dicyclopentyl-6-methyl-	14.9305	6.3364
38	Allonic acid, .gamma.-lactone, 4TMS derivavative	15.2510	0.0988
*6. Hydrazides and Oxo-Acids*			
10	2-Oxopentanoic acid, TMS derivative	11.6175	0.0526
11	2-(2-Bromo-4-methylphenoxy)-N’-([1-(4-nitrophenyl)-2-pyrrolidinyl]methylene)acethydrazide	11.7663	3.3904
13	2-Oxopentanoic acid, TMS derivative	12.3843	0.0495
16	2-Oxopentanoic acid, TMS derivative	12.6246	0.0482
40	3-tert-Butyl-N’-(3-phenyl-2-propenylidene)-1H-pyrazole-5-carbohydrazide ditms	15.5256	0.0712
*7. Other Lipids and Alcohols*			
1	2-Methyl-1,4-bis(trimethylsiloxy)butane	8.3846	0.0195
17	9-Cycloheptadecen-1-ol	12.9450	16.0008
23	Acetin, bis-1,3-trimethylsilyl ether	14.1409	0.0488
25	1-Monopalmitin, 2TMS derivative	14.2840	1.0110

RT: Retention time; TMS: Trimethylsilyl; TBDMS: *tert*-Butyldimethylsilyl. Data obtained via GC–MS analysis. The area % represents the relative abundance of each compound based on the total integrated peak area. Compounds were analyzed as silylated derivatives to improve volatility and separation.

### Characterization of selenium nanoparticles (ME-SeNPs)

**Fig. 3:**
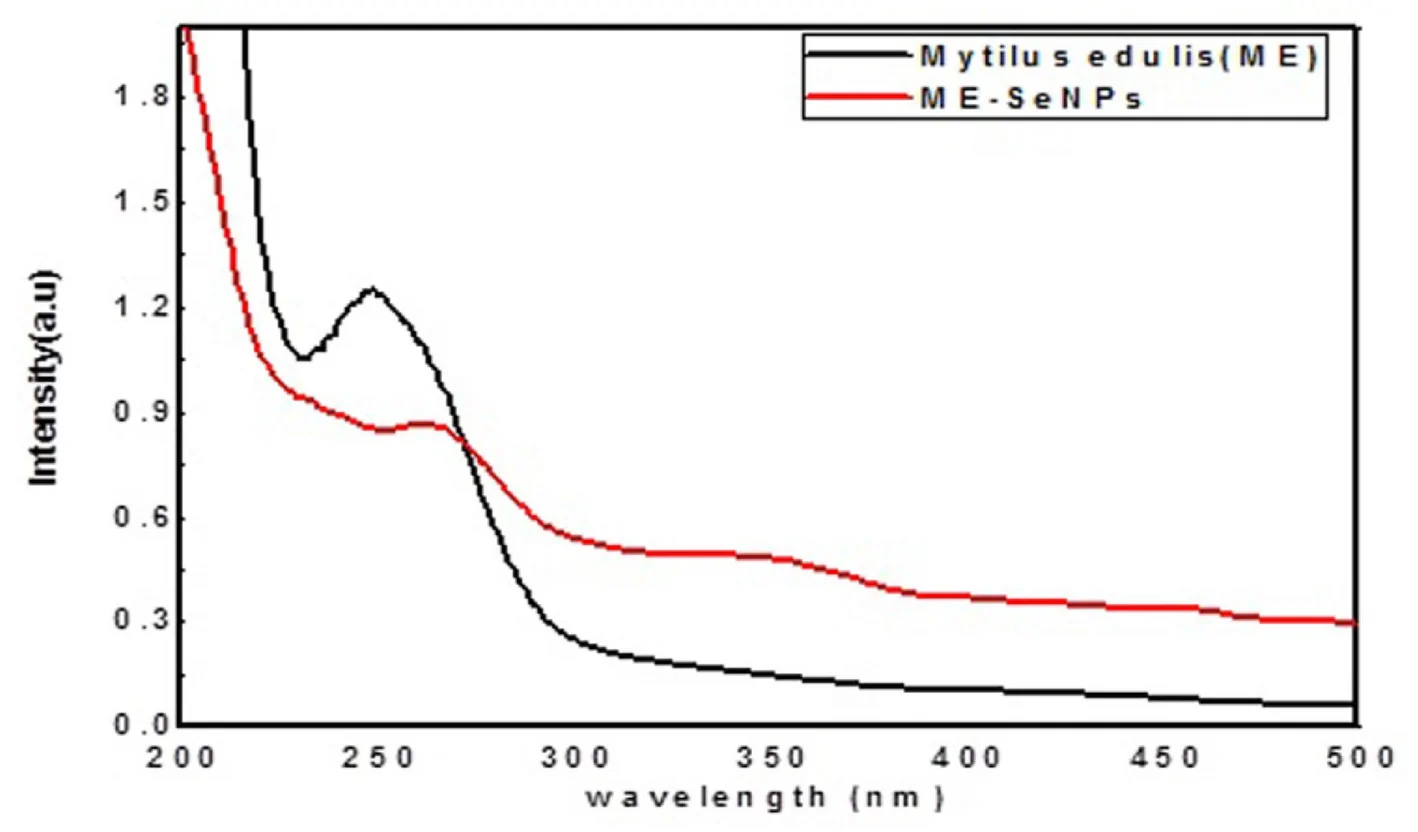
UV–Visible absorption spectra of *Mytilus edulis* (ME) extract and synthesized ME-mediated selenium nanoparticles (ME-SeNPs).

**Fig. 4:**
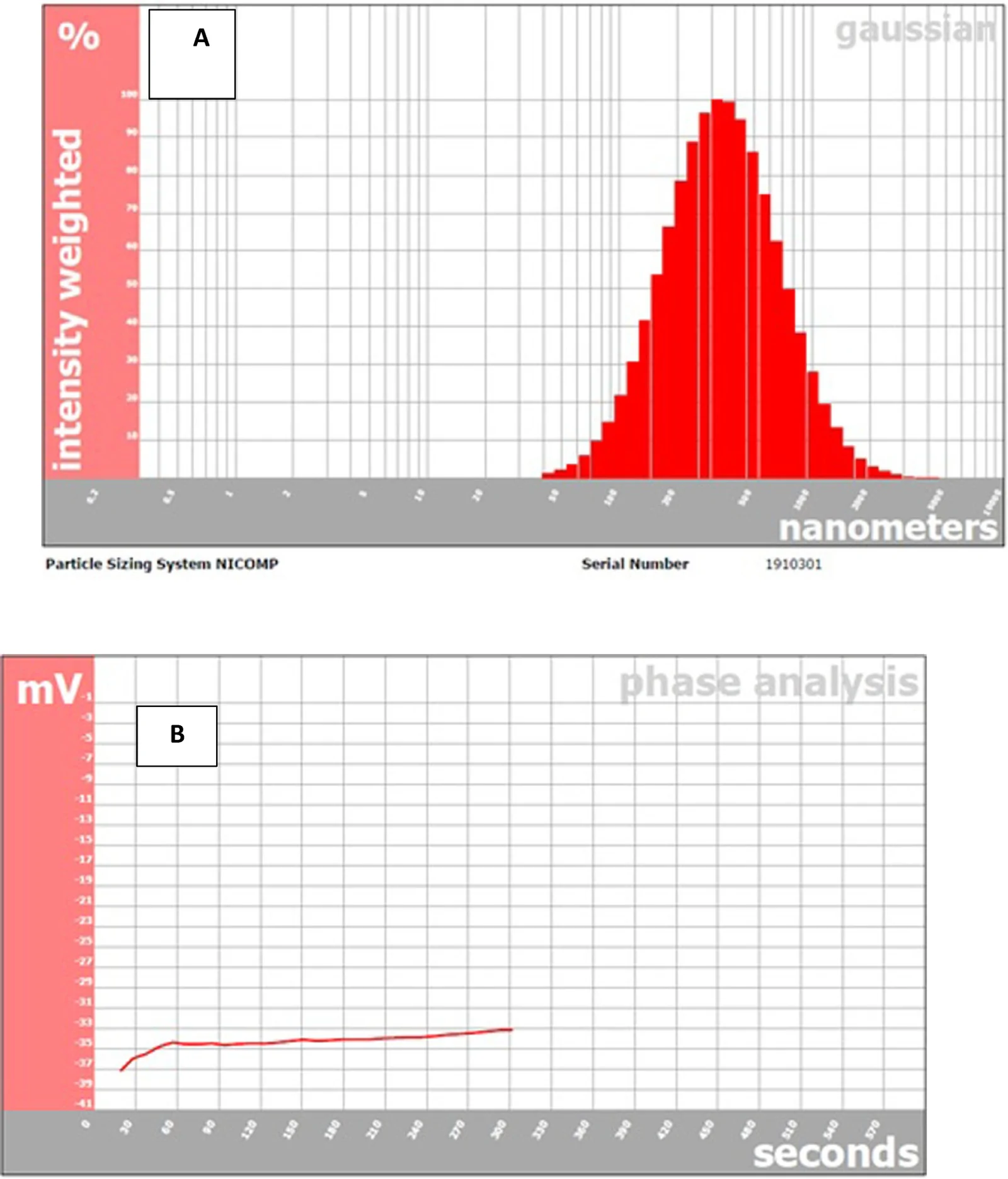
(**A**) Particle size distribution of *Mytilus edulis* -mediated selenium nanoparticles (ME-SeNPs); (**B**) zeta potential analysis.

UV–Visible spectroscopy was used to monitor the synthesis and optical properties of *Mytilus edulis*-mediated selenium nanoparticles (ME-SeNPs). The crude extract of *M. edulis* exhibited ultraviolet absorption near 250 nm (Fig. 3), corresponding to endogenous bioactive constituents such as proteins and peptides involved in the reduction process. Upon nanoparticle formation, an absorption band emerged at approximately 280 nm, characteristic of elemental selenium Se^0^synthesis. This conversion was accompanied by a color transition from pale yellow to ruby red/brownish-orange^61^. Dynamic Light Scattering (DLS) analysis showed that the ME-SeNPs had an average hydrodynamic diameter of 292 nm with a polydispersity index (PDI) of 0.47 (Fig. 4a). The zeta potential was measured at − 33.13 mV (Fig. 4b), indicating moderate size distribution and electrostatic colloidal stability. The net negative charge provides electrostatic repulsion between particles, helping to prevent rapid aggregation in dispersion.

**Fig. 5:**
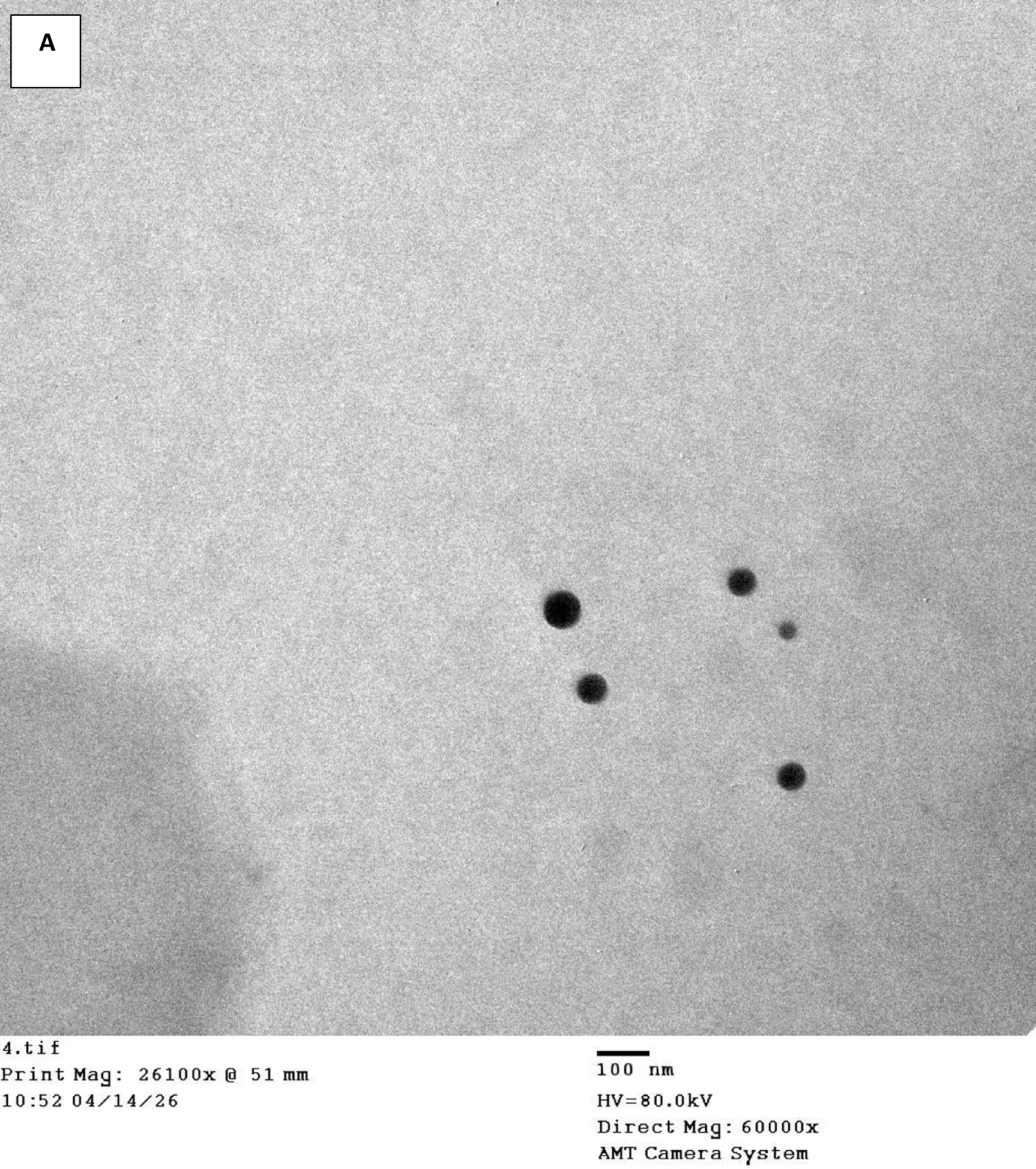

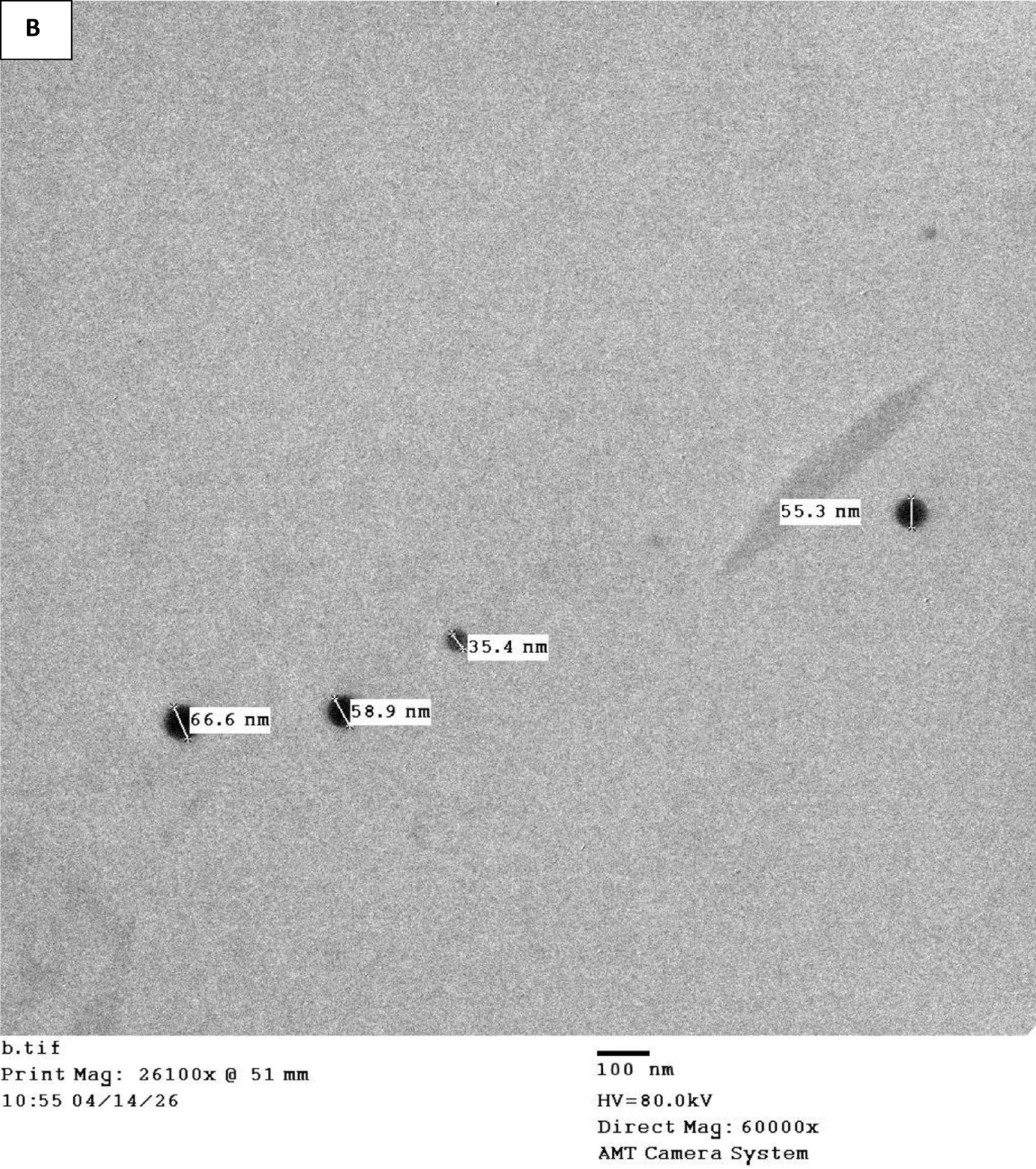

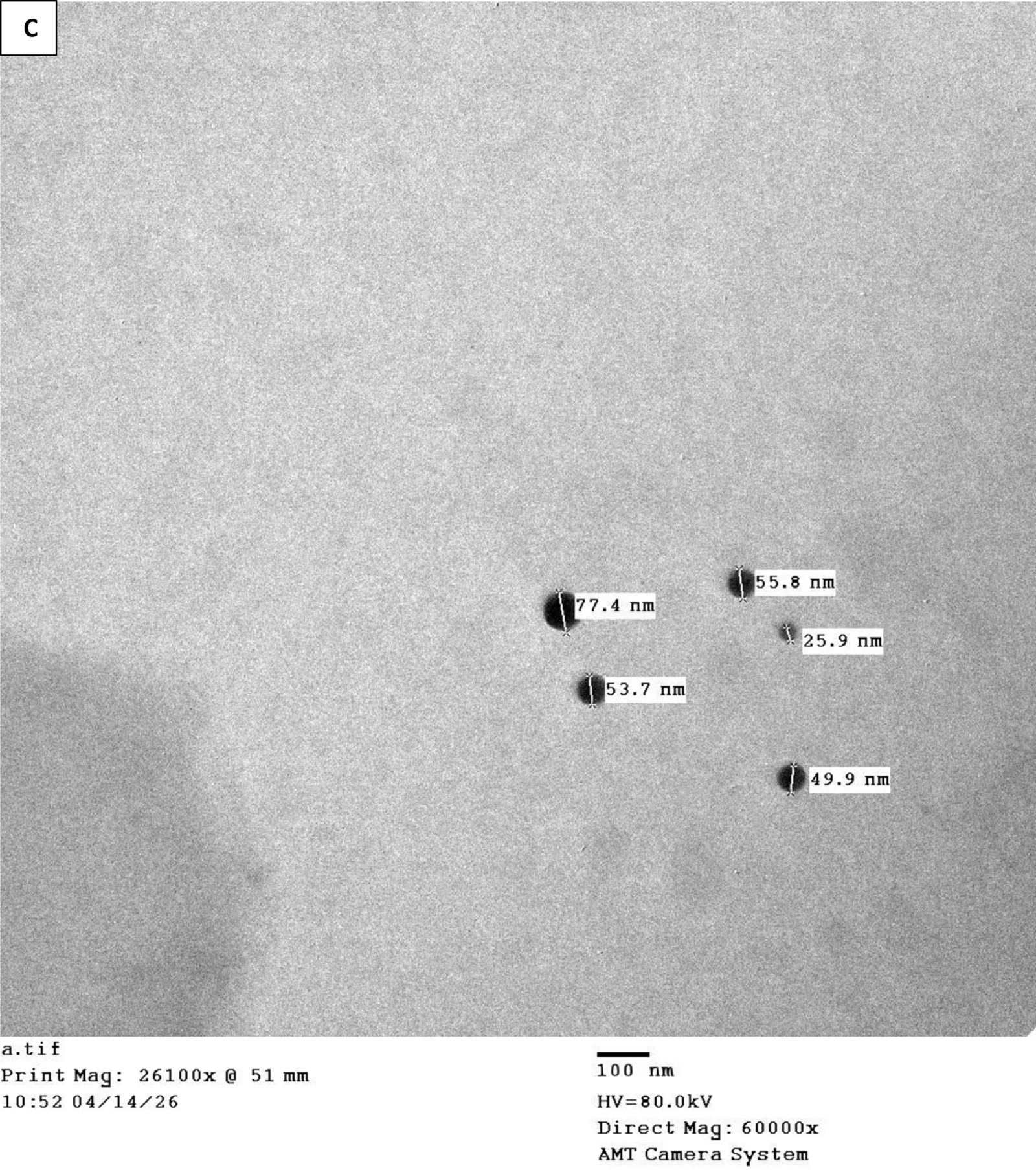
The TEM images in panels A, B, and C show the morphology and size distribution of *Mytilus edulis*–mediated selenium nanoparticles (ME-SeNPs).

Transmission Electron Microscopy (TEM) analysis (Fig. 5B, C) demonstrated that the ME-SeNPs were predominantly spherical and uniformly distributed, with particle sizes ranging from 35 to 65 nm. This spherical morphology is favorable for enhanced dispersion and reduced aggregation behavior^62^.

**Fig. 6:**
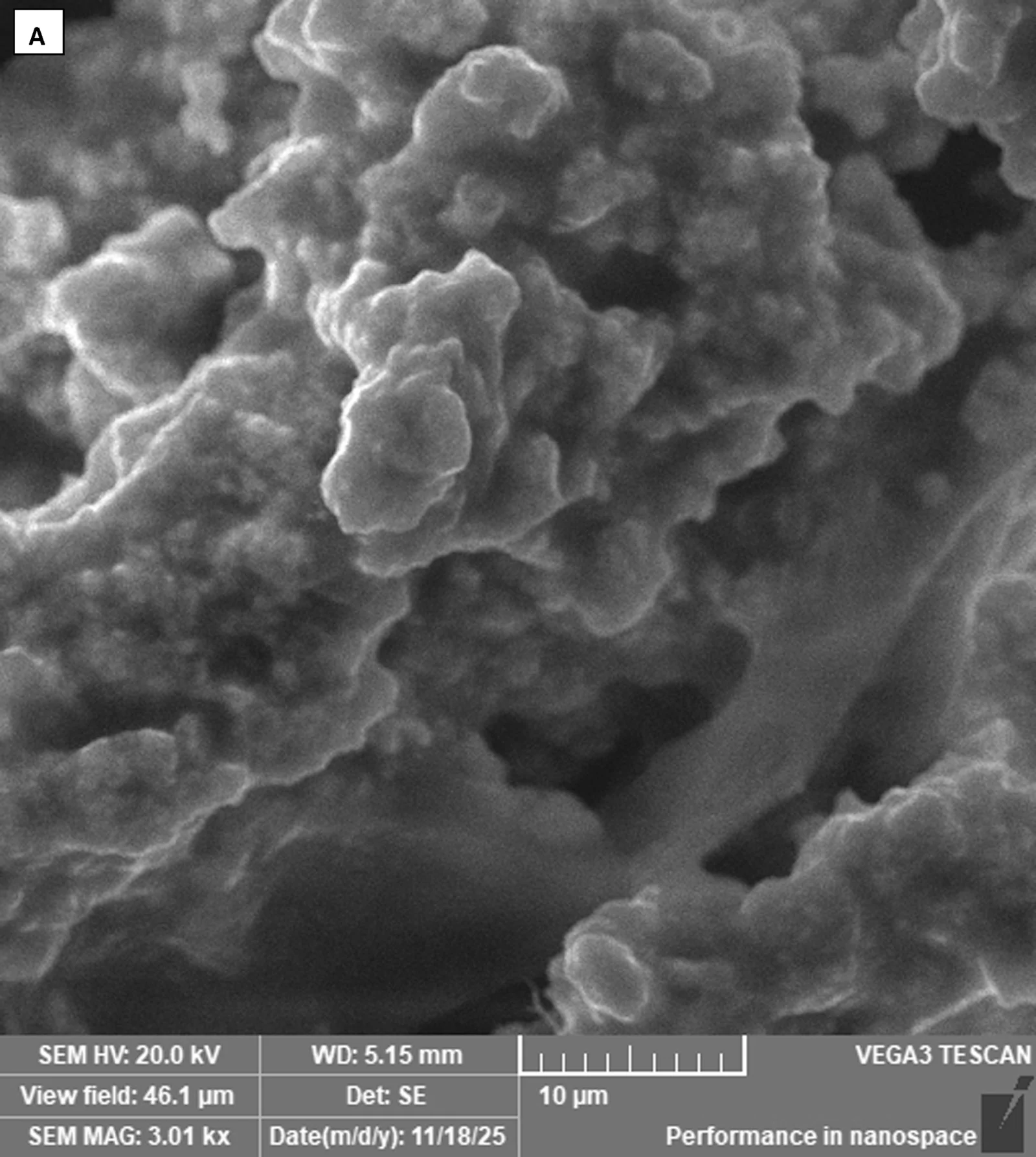

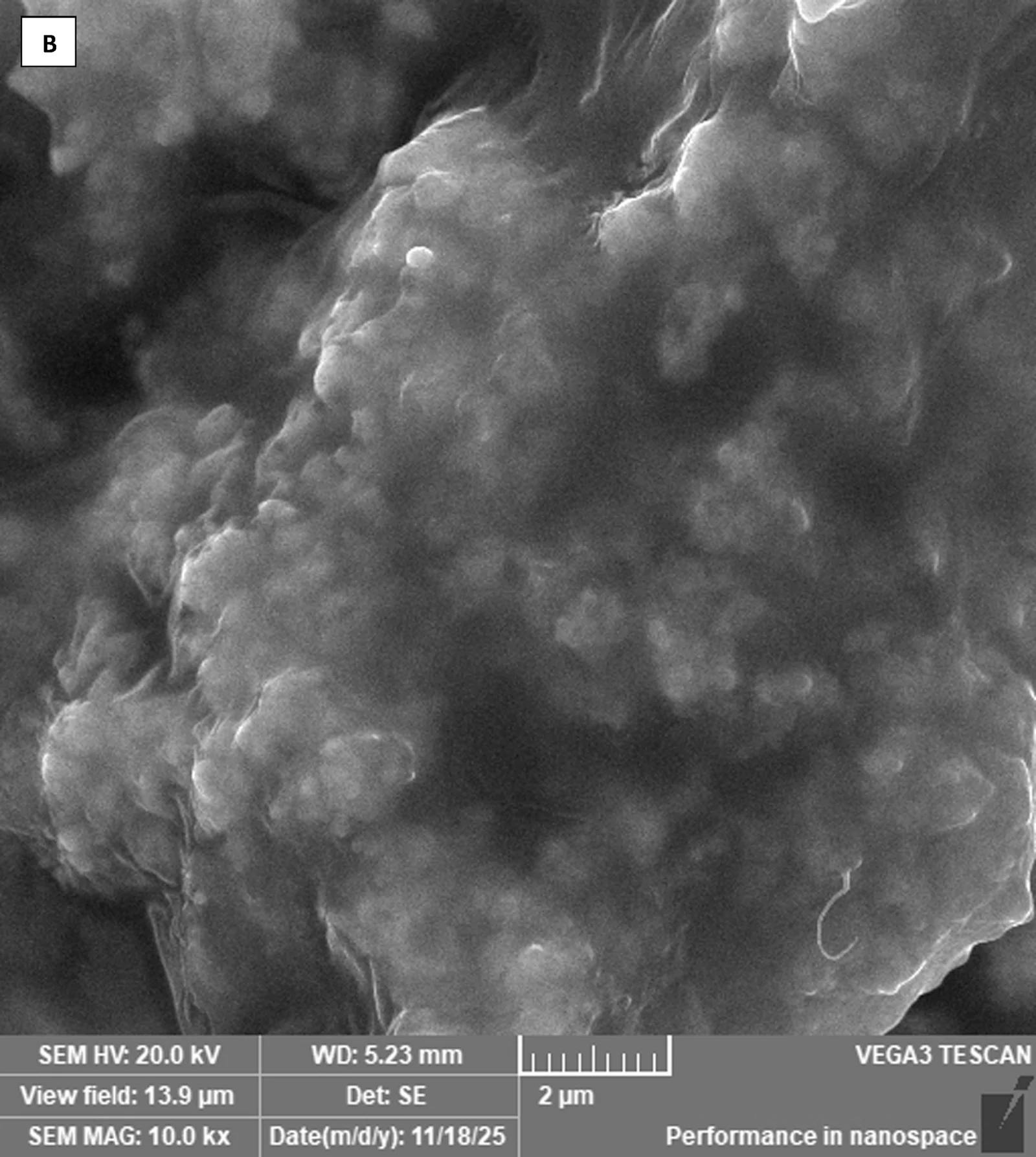
Scanning electron microscopy (SEM) images of *Mytilus edulis*–mediated selenium nanoparticles (ME-SeNPs) in panels A and B at different magnifications (10 µm and 2 µm), illustrating the surface morphology and structural features.

Scanning Electron Microscopy (SEM) (Fig. 6A & B) further confirmed the surface morphology of the biosynthesized nanoparticles, revealing well-dispersed spherical structures with smooth surfaces, consistent with successful green synthesis.

**Fig. 7:**
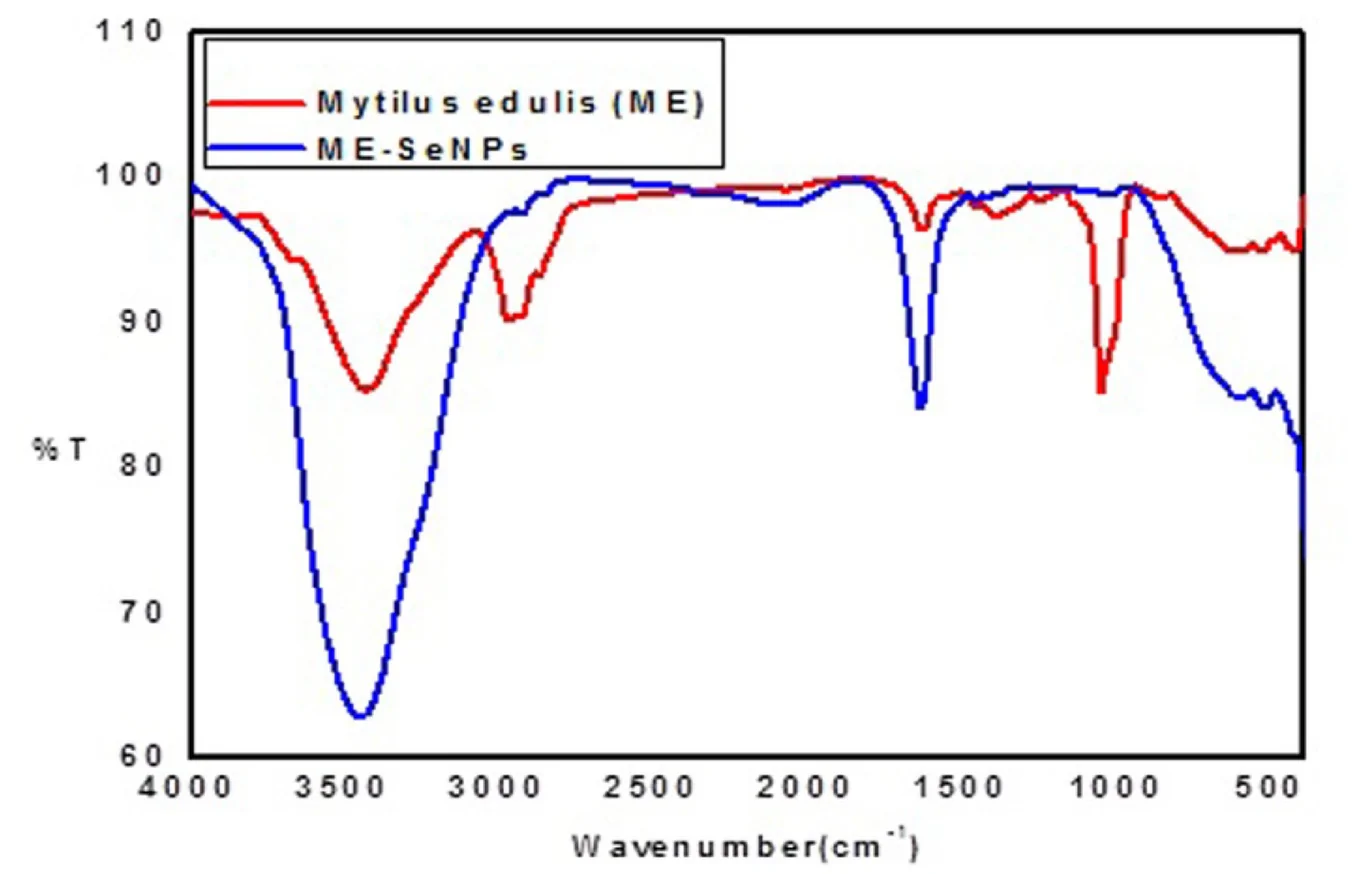
Fourier Transform Infrared (FTIR) spectra of *Mytilus edulis* (ME) extract and *Mytilus edulis* -mediated selenium nanoparticles (ME-SeNPs), illustrating functional group involvement in nanoparticle synthesis and stabilization.

Fourier Transform Infrared (FTIR) spectroscopy (Fig. 7) was used to identify the functional groups involved in nanoparticle formation. The FTIR spectrum of the *M. edulis* extract showed characteristic peaks at 3428 cm^−1^ (N–H stretching, amides), 1633 cm^−1^ (C=O stretching), and 1455 cm^−1^ and 854 cm^−1^ (C–O stretching of carbonate), indicating the presence of proteins and carbonate-containing biomolecules. Additional bands at 2969 cm^−1^ (amide B of α-chitin) and within the range of 1100–1700 cm^−1^ (amide and chitin-related vibrations) were also observed^63,64^.

In contrast, the FTIR spectrum of ME-SeNPs displayed shifted and modified peaks at 3441, 2924, 2856, and 1639 cm^−1^, corresponding to O–H stretching, C–H vibrations, and amide-related functional groups.^65^ Additional peaks at 1372 cm^−1^, 1240 cm^−1^, and 1101 cm^−1^ were attributed to polysaccharide and carboxyl-related vibrations^66^. Notably, significant spectral shifts were observed, including red shifts from 1455 to 1459 cm^−1^, 3428 to 3446 cm^−1^, and 1633 to 1638 cm^−1^, the disappearance of the 854 cm^−1^ band, and a blue shift from 2969 to 2924 cm^−1^. These changes may confirm the active involvement of biomolecules from the *M. edulis* extract in the reduction, capping, and stabilization of selenium nanoparticles.

### Fourier Transform Infrared (FTIR) spectroscopy analysis and biomolecular surface functionalization of ME-SeNPs

The FTIR spectra of both the *Mytilus edulis* extract (ME) and the synthesized selenium nanoparticles (ME-SeNPs) Table 3 and Fig. 7 reveal distinct vibrational shifts, confirming the active role of biomolecules in the reduction and capping processes. The shift in the O–H/N–H stretching region (~ 3428 to ~ 3441^–1^ cm) along with changes in the Amide I and II bands (~ 1633 to ~ 1638^–1^ cm and ~ 1540 to ~ 1528^–1^ cm, respectively) underscore the participation of proteins and polyphenols in stabilizing the nanoparticles through carbonyl coordination and hydrogen bonding. Furthermore, the shifts in C–H (~ 2969 to ~ 2924^–1^ cm) and C–O/C–O–C functional groups imply that lipids and structural polysaccharides form a robust hydrophobic and electrostatic capping layer. Crucially, the emergence and shift of the band at the lower frequency region (~ 615 to ~ 598^–1^ cm) validates the successful formation of biogenic SeNPs and confirms direct Se–biomolecule surface bonding.

**Table 3:** Fourier Transform Infrared (FTIR) spectroscopy absorption bands and corresponding functional group shifts between *Mytilus edulis* extract (ME) and biogenic selenium nanoparticles (ME-SeNPs).

Wavenumber in ME (cm^−1^)	Wavenumber in ME SeNPs (cm^−1^)	Assignment	Interpretation
~ 3428	~ 3441	O–H / N–H stretching (polyphenols, proteins)	H-bonding with SeNP surface; hydroxyl coordination
~ 2969	~ 2924	C–H asymmetric stretching (lipids, aliphatic chains)	Hydrophobic association with capping layer
~ 1633	~ 1638	C=O stretching / Amide I (proteins)	Carbonyl coordination to Se surface
~ 1540	~ 1528	N–H bending / Amide II (proteins)	Conformational change upon adsorption
~ 1380	~ 1365	C–O stretching / carboxylate (–COO^−^)	Electrostatic/covalent binding to SeNPs
~ 1250	~ 1240	C–O–C / C–N stretching (polysaccharides, amines)	Surface functionalization by polysaccharides
~ 1045	~ 1032	C–O stretching (alcohols, polyphenols)	Phenolic –OH involvement in Se^4^⁺ reduction
~ 615	~ 598	Se–O or Se–C stretching	Direct Se–biomolecule bonding at surface

Wavenumbers are expressed in reciprocal centimeters (cm^−1^) and preceded by the tilde symbol ( ~) to denote approximate peak positions obtained via FTIR spectroscopy. ME represents the raw biochemical matrix of *Mytilus edulis*, while ME SeNPs denotes the biogenic selenium nanoparticles synthesized and stabilized using this marine extract.

### Screening of antimicrobial activity of selenium nanoparticles (ME-SeNPs)

**Fig. 8:**
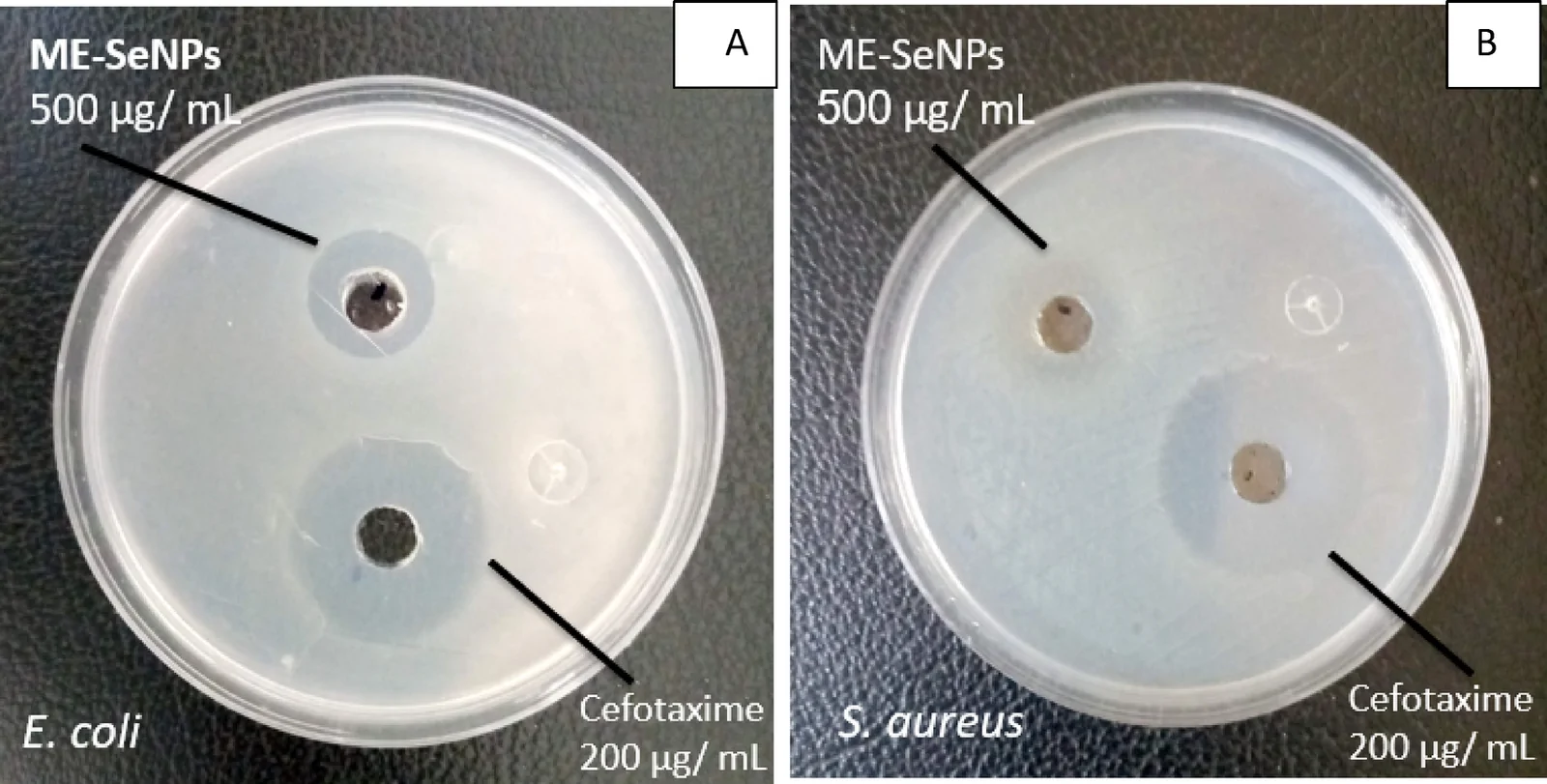
(**A**, **B**): Antimicrobial activity of Selenium Nanoparticles (ME-SeNPs) (500 µg/mL) showing inhibition zones against *S. aureus* ATCC 25,923 *and E, coli* ATCC 25,922 compared to cefotaxime (200 µg/ mL) as a reference antibiotic. Data are expressed as mean ± standard deviation (SD) from three independent experiments (n = 3).

As shown in Fig. 8A, B, Selenium Nanoparticles (ME-SeNPs) (500 µg/ mL) showed a moderate clear inhibition zones against both *S. aureus* ATCC 25923 strain (13 ± 0.43 mm) and *E. coli* ATCC 25922 (15 ± 0.58 mm), whereas cefotaxime (200 µg/ mL) is significantly more effective with zones of inhibition exceeding 22 ± 0.30 mm.

### The minimum inhibitory concentration (MIC) of selenium nanoparticles (ME-SeNPs) for *Staphylococcus aureus *ATCC 25923 and *Escherichia coli* ATCC 25922

**Fig. 9:**
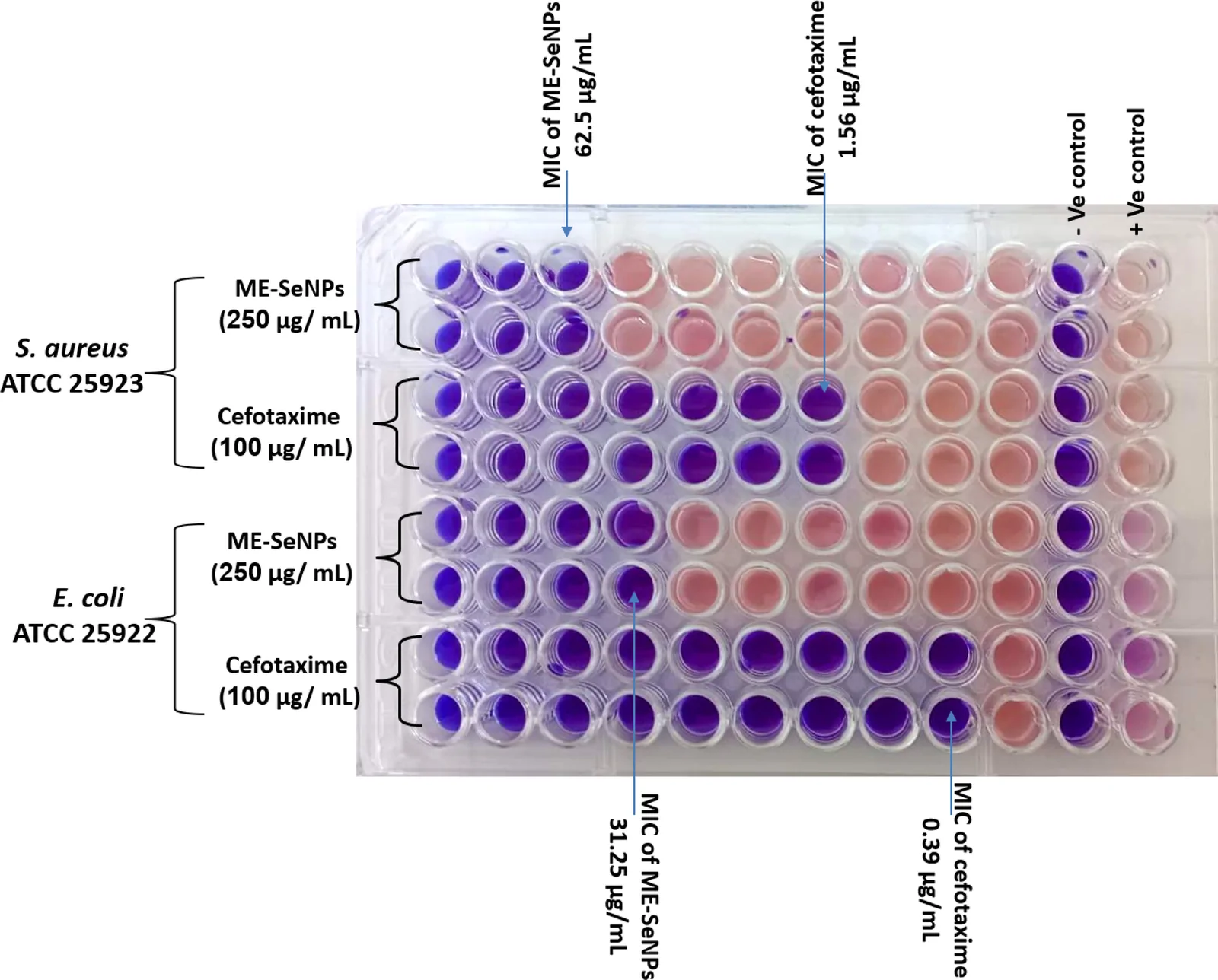
MIC of Selenium Nanoparticles (ME-SeNPs) and cefotaxime against *Staphylococcus aureus ATCC 25923 and Escherichia coli ATCC 25922* strains.

The antibacterial efficacy of the biogenic ME-SeNPs was evaluated against *S. aureus* ATCC 25923 and *E. coli* ATCC 25922, using the commercial antibiotic cefotaxime as a positive control (Table 4, Fig. 9). When compared on an equivalent mass basis (µg/mL), the green-synthesized nanoparticles demonstrated potent antimicrobial activity, displaying a more pronounced inhibitory effect against the Gram-negative strain than the Gram-positive one. Specifically, for *E. coli* ATCC 25922, the ME-SeNPs exhibited a minimum inhibitory concentration (MIC) of 31.25 ± 0.0 µg/mL whereas the MIC for *S. aureus* ATCC 25923 was two-fold higher at 62.5 ± 0.0 µg/mL In comparison, cefotaxime yielded MIC values of 0.39 ± 0.0 µg/mL and 1.56 ± 0.0 µg/mL against *E. coli* and *S. aureus*, respectively. These findings underscore the competitive broad-spectrum bactericidal potential of ME-SeNPs, with a distinct susceptibility favored toward Gram-negative bacterial profiles.

**Table 4:** Minimum Inhibitory Concentration (MIC) values (µg/ mL) of Selenium Nanoparticles (ME-SeNPs) and cefotaxime against *Staphylococcus aureus ATCC 25923 and Escherichia coli ATCC 25922*

Strain	MIC (µg/ mL)	
	ME-SeNPs	Cefotaxime
*S. aureus* ATCC 25923	62.5 ± 0.0	1.56 ± 0.0
*E. coli* ATCC 25922	31.25 ± 0.0	0.39 ± 0.0

Data are expressed as mean ± standard deviation (SD) from three independent experiments (n = 3).

### Time-kill assay of selenium nanoparticles (ME-SeNPs) against *Staphylococcus aureus ATCC 25923 and Escherichia coli ATCC 25922* strains

**Fig. 10:**
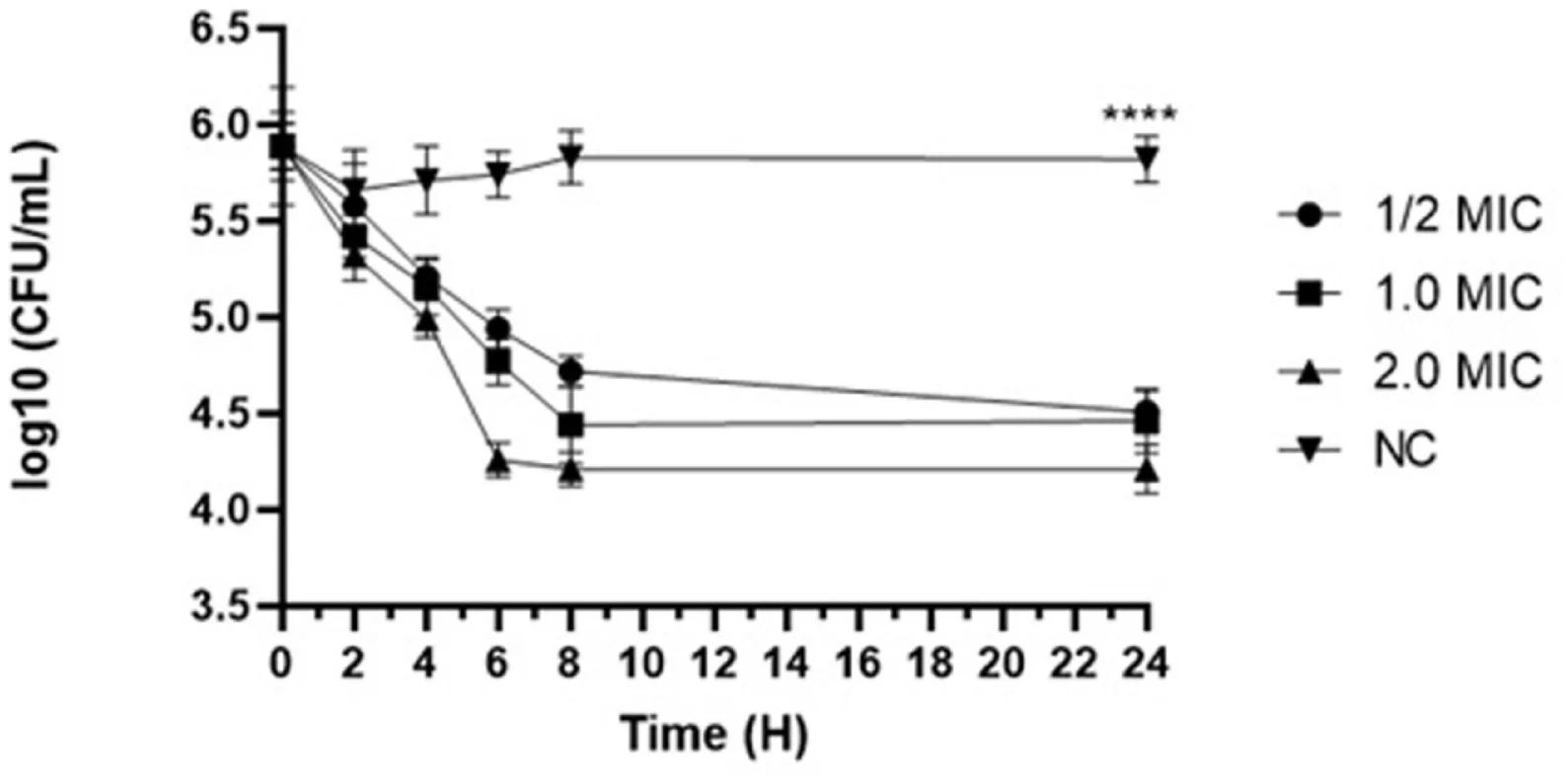
Time kill assay of Selenium Nanoparticles (ME-SeNPs) against* S. aureus* ATCC 25923. Data points represent the mean ± SD (n = 3). *Statistical significance compared to the untreated control group is indicated by asterisks p* < *0.001, determined by Two-way ANOVA followed by Dunnett’s post-hoc test.*

The time-kill kinetics assay was performed to evaluate the dynamic antimicrobial activity of selenium nanoparticles (ME-SeNPs) against *Staphylococcus aureus* ATCC 25923. At 0 h, the starting bacterial inoculum across all experimental groups was stable at an average of 5.89 ± 0.12 to 0.31log_10_ CFU/mL. Following exposure to ME-SeNPs, a clear concentration-dependent reduction in bacterial viability was observed over time. Specifically, within the first 8 h of incubation, the bacterial counts progressively decreased across the sub-MIC and MIC treatment. By 24 h, the final log reductions achieved were 1.38log_10_ CFU/mL for the 0.5 MIC group (4.51 ± 0.11log_10_ CFU/mL), 1.43log10 CFU/mL for the 1.0 MIC group (4.46 ± 0.17 log_10_ CFU/mL), and a maximum reduction of 1.68log_10_ CFU/mL for the 2.0MIC group (4.21 ± 0.13log_10_ CFU/mL). In contrast, the untreated negative control (NC) group maintained a stable active load fluctuating around 5.82 ± 0.12log_10_ CFU/mL. Because the maximum decrease at 24 h remained below the standard 3.0log_10_ CFU/mL (99.9% killing) threshold, the effect does not meet the criteria for bactericidal action and is classified as predominantly bacteriostatic against this Gram-positive strain. Statistical validation via two-way ANOVA followed by Dunnett’s multiple comparisons post-hoc test confirmed that the observed reductions at all treatment concentrations (0.5, 1.0 and 2.0 MIC) from the 2-h mark through the 24-h endpoint were extremely significant compared to the untreated control group (*p* < 0.0001) (Table 5, Fig. 10).

**Table 5:** Time kill assay of Selenium Nanoparticles (ME-SeNPs) against *Staphylococcus aureus* ATCC 25923 and *Escherichia coli* ATCC 25922 strains

Selenium Nanoparticles (ME-SeNPs)/ *S. aureus* ATCC 25,923				
	Log cfu ± SD			
Time (H)	1/2 MIC	1.0 MIC	2.0 MIC	NC
0 H	5.89 ± 0.31	5.89 ± 0.12	5.89 ± 0.18	5.89 ± 0.12
2 H	5.58 ± 0.22	5.42 ± 0.11	5.32 ± 0.13	5.66 ± 0.21
4 H	5.21 ± 0.09	5.16 ± 0.15	4.99 ± 0.10	5.71 ± 0.18
6 H	4.94 ± 0.10	4.77 ± 0.12	4.26 ± 0.09	5.74 ± 0.12
8 H	4.72 ± 0.08	4.44 ± 0.20	4.21 ± 0.09	5.83 ± 0.14
24 H	4.51 ± 0.11	4.46 ± 0.17	4.21 ± 0.13	5.82 ± 0.12

Data are expressed as mean ± standard deviation (SD) from three independent experiments (n = 3)

The time-kill kinetics assay revealed that selenium nanoparticles (ME-SeNPs) exerted a dose-dependent bactericidal action against the Gram-negative strain *Escherichia coli* ATCC 25922. At 0 h, the baseline for bacterial inoculum across all experimental setups was uniformly stabilized at approximately 5.56 ± 0.0.9 to 0.24 log_10_ CFU/mL. Exposure to ME-SeNPs triggered a sharp and progressive decrease in viable bacterial cells over the course of the 24-h incubation period, presenting a notable concentration-dependent relationship.

Specifically, treatment at the sub-inhibitory threshold (0.5 MIC) showed a steady reduction trend, dropping to 3.39 ± 0.14log_10_ CFU/mL at 8 h and concluding at 2.61 ± 0.11log_10_ CFU/mL after 24 h. More pronounced killing speeds were observed at the inhibitory concentrations; the 1.0 MIC group experienced a distinct down-slope, reaching 2.05 ± 0.15 log_10_ CFU/mL at 8 h and falling to a final viable count of 1.56 ± 0.12 log_10_ CFU/mL at 24 h. The most intense antimicrobial activity was documented at 2.0 MIC, where viable counts steeply declined to near-baseline levels, tracking at 1.35 ± 0.10 log ± _10_ CFU/mL at 8 h and hitting a final endpoint of 1.04 ± 0.23log10 CFU/mL at 24 h. In contrast, the untreated negative control (NC) group showed typical active logarithmic multiplication, expanding from the initial inoculum up to 7.23 ± 0.20 log_10_ CFU/mL at the 24-h benchmark.

**Fig. 11:**
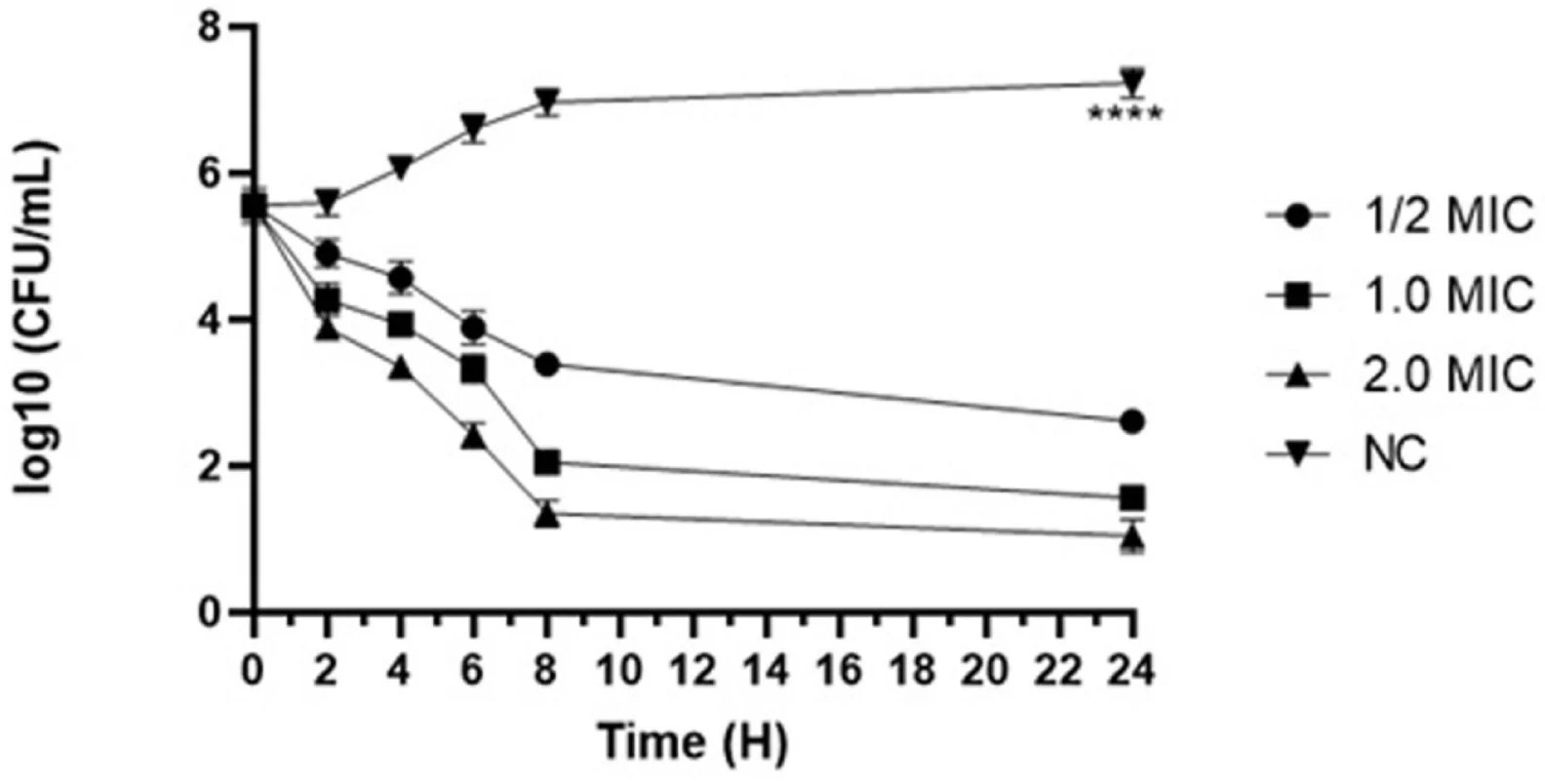
Time kill assay of Selenium Nanoparticles (ME-SeNPs) against *E. coli* ATCC 25922. Data points represent the mean ± SD (n = 3). Statistical significance compared to the untreated control group is indicated by asterisks* p < *0.001, determined by Two-way ANOVA followed by Dunnett’s post-hoc test.*

When calculating the total net impact at 24 h, the 1.0 MIC and 2.0 MIC regimens achieved final reductions of 4.0 log_10_ CFU/mL and 4.52 log10CFU/mL, respectively, relative to the initial starting load. Because these values significantly exceed the standard epidemiological benchmark of a ≥ log 3.0 CFU/mL reduction (equivalent to a 99.9% killing velocity), the dynamic performance of ME-SeNPs meets the formal criteria to be classified as a true bactericidal agent against *E. coli*. Statistical validation of these kinetics via a two-way ANOVA followed by Dunnett’s multiple comparisons test confirmed that while baseline variance at 0 h was completely non-significant *p* > 0.9999), the observed microbial drops for all treated concentrations (0.5, 1.0, and 2.0 MIC) from the 2-h mark through to the 24-h endpoint were extremely significant compared to the actively growing control group (*p* < 0.0001) (Table 6, Fig. 11).

**Table 6:** Time kill assay of Selenium Nanoparticles (ME-SeNPs) against *E. coli* ATCC 25922

Selenium Nanoparticles ME-SeNPs / *E. coli* ATCC 25922				
	Log cfu Log cfu ± SD			
Time (H)	1/2 MIC	1.0 MIC	2.0 MIC	NC
0 H	5.56 ± 0.15	5.56 ± 0.24	5.56 ± 0.09	5.56 ± 0.12
2 H	4.90 ± 0.19	4.27 ± 0.22	3.89 ± 0.12	5.60 ± 0.18
4 H	4.57 ± 0.22	3.94 ± 0.16	3.36 ± 0.15	6.07 ± 0.11
6 H	3.89 ± 0.23	3.33 ± 0.19	2.42 ± 0.17	6.61 ± 0.19
8 H	3.39 ± 0.14	2.05 ± 0.15	1.35 ± 0.10	6.97 ± 0.18
24 H	2.61 ± 0.11	1.56 ± 0.12	1.04 ± 0.23	7.23 ± 0.20

## Molluscicide of selenium nanoparticles (ME-SeNPs) against *Biomphalaria alexandrina* snails

The results presented in Table 7 demonstrate that the biogenic selenium nanoparticles (ME-SeNPs) exhibited a clear dose-dependent molluscicidal activity against *Biomphalaria alexandrina* snails. Probit regression analysis performed via SPSS yielded LC 10, LC 25, LC 50, and LC 90 values of 86.89, 110.06, 135.27, and 184.48 mg/L, respectively. The median lethal concentration (LC 50) was established at 135.27 mg/L, with 95% confidence intervals spanning from 96.10 to 161.86 mg/L, while the LC 90 value was determined to be 184.48 mg/L (95% CI 159.31–301.70 mg/L). Furthermore, the calculated regression slope of 1.18 reflects a highly homogenous population response, indicating a consistent, predictable, and reliable susceptibility of the targeted snails to the nanoparticle treatment.

**Table 7:** Molluscicide activity of Selenium Nanoparticles (ME-SeNPs) Against *Biomphalaria alexandrina* snails.

Selenium (ME-SeNPs)	LC_10_ mg/L	LC_25_ mg/L	LC_50_ mg/L	LC_90_ mg/L	Slope
24 h Exposure followed 24 h recovery	86.89	110.06	135.72 (96.10–161.86) Confidence limits	184.48 (159.31–301.70) Confidence limits	1.18

LC 10, LC 25, LC 50, and LC 90 represent the lethal concentrations required to kill 10%, 25%, 50%, and 90% of *Biomphalaria alexandrina* snails, respectively. Values in parentheses indicates the 95% confidence limits (CL). Slope values represent the steepness of the dose–response curve. All toxicity values were calculated based on Probit analysis.

### Impact of Selenium Nanoparticles (ME-SeNPs) on hemocyte transformation in *Biomphalaria alexandrina* snails

**Fig. 12:**
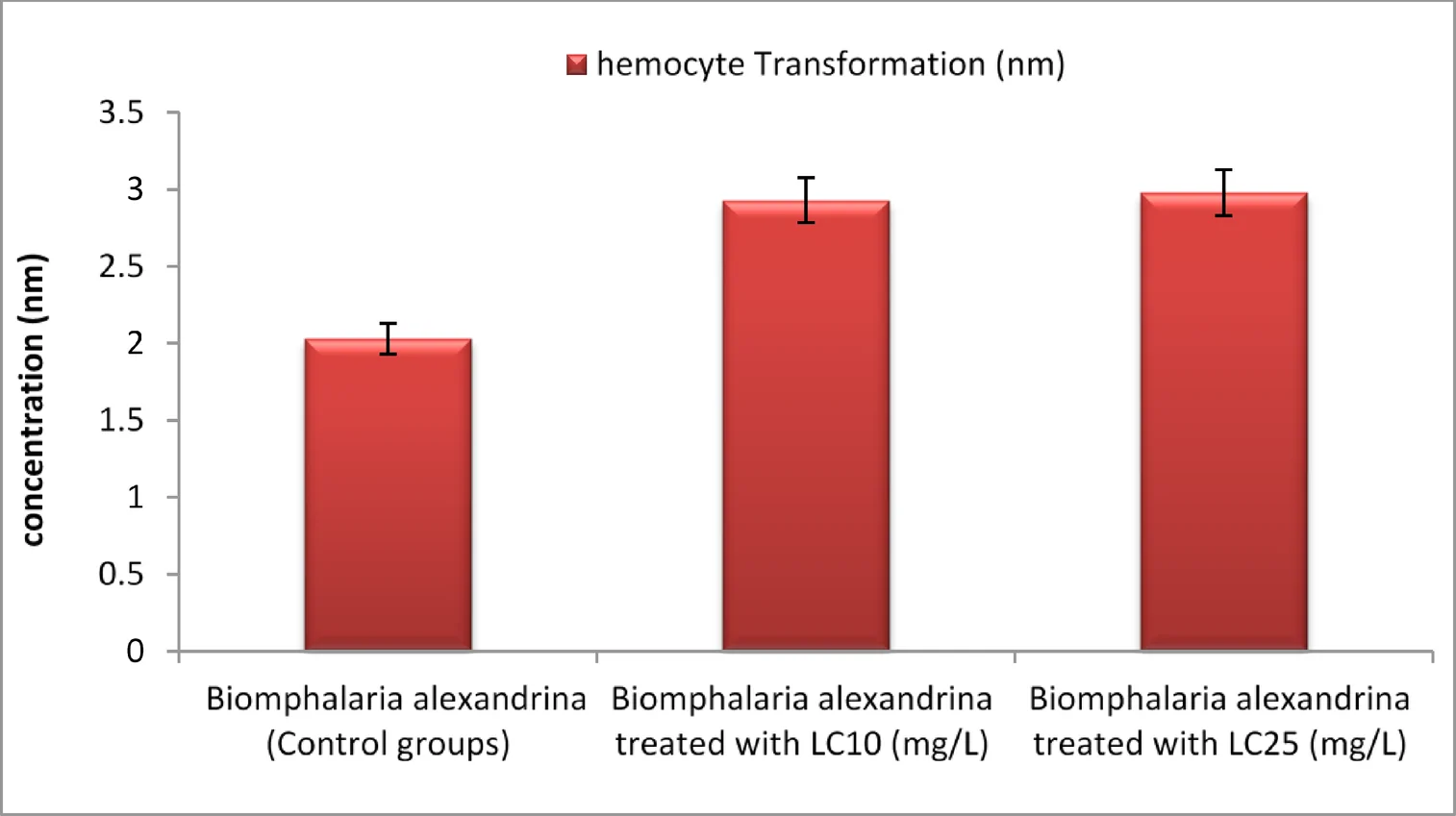
Effect of Selenium Nanoparticles (ME-SeNPs) on *Biomphalaria alexandrina* snils on hemocyte Transformation in *Biomphalaria alexandrina* snails. Data are expressed as mean ± SD. Statistical analysis was performed using one-way ANOVA. Different letters indicate significant differences among treatments at *p* < 0.05.

The effects of sublethal exposure to ME-SeNPs on the cellular immune response of *Biomphalaria alexandrina* were evaluated using the hemocyte transformation assay (n = 3 independent replicates per group; data expressed as mean ± SD). As compiled in Table 8 and Fig. 12, the baseline hemocyte proliferation in the control group exhibited an optical density (OD) of 2.03 ± 0.54.Exposure to the sublethal LC 10 concentration of ME-SeNPs for 24 h, followed by a 24-h recovery period, induced a statistically significant elevation in proliferation absorbance to 2.93 ± 0.42(One-way ANOVA, *p* < 0.05). Similarly, snails exposed to the higher sublethal LC 25 threshold exhibited a further significant increase in proliferation, reaching an absorbance value of 2.98 ± 0.23 (*p* < 0.05).

**Table 8:** Effect of Selenium Nanoparticles (ME-SeNPs) on hemocyte transformation in absorbance (nm) in *Biomphalaria alexandrina* snails.

Treatments groups	Hemocyte transformation in absorbance (nm)
*Biomphalaria alexandrina* (Control groups)	2.03 ± 0.54
*Biomphalaria alexandrina* treated with LC10 (mg/L) of (ME-SeNPs)for 24 h then recovered for 24 h	2.93 ± 0.42
*Biomphalaria alexandrina* treated with LC_25_(mg/L) (ME-SeNPs) for 24 h then recovered for 24 h	2.98 ± 0.23

Data are expressed as mean ± SD (n = 3 independent replicates per group). After verifying data normality and homogeneity of variance, statistical significance was determined using One-way ANOVA. Differences were considered statistically significant at *p* < 0.05 compared with the control group.

### Impact of Selenium Nanoparticles (ME-SeNPs) on nitric oxide (NO) concentration in *Biomphalaria alexandrina* snails

**Fig. 13:**
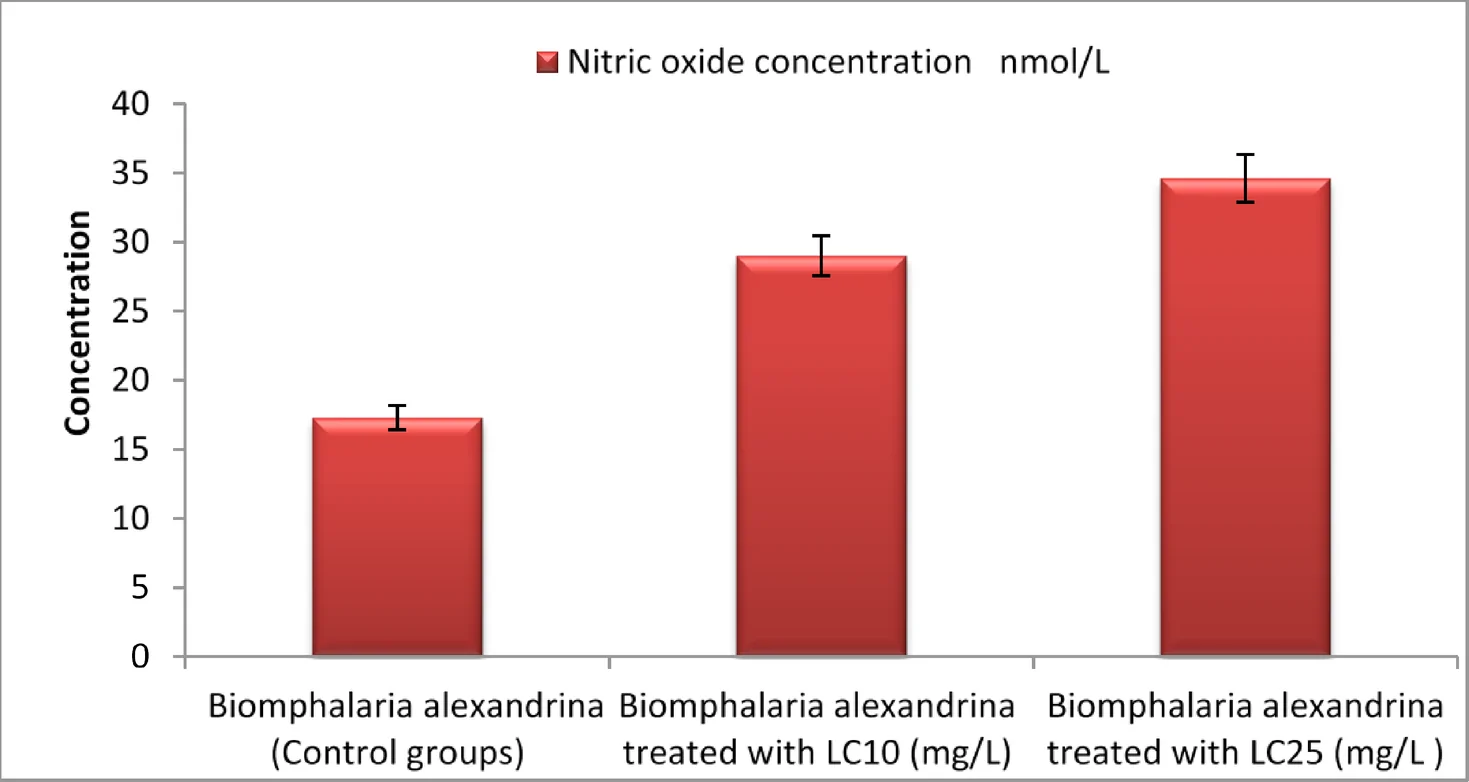
Effect of Selenium Nanoparticles (ME-SeNPs) on nitric oxide (NO) levels in *Biomphalaria alexandrina* snails homogenized tissues. Data are expressed as mean ± SD. Statistical analysis was performed using one-way ANOVA. Different letters indicate significant differences among treatments at *p* < 0.05.

The quantitative impact of sublethal ME-SeNPs exposure on nitrosative stress biomarkers in *Biomphalaria alexandrina* was determined by measuring intracellular nitric oxide (NO) concentrations (n = 3 independent replicates per group; data expressed as mean ± SD). As summarized in Table 9 and Fig. 13 the baseline nitric oxide concentration in the control group was maintained at 17.29 ± 0.96 nmol/L. Following a 24-h exposure to the sublethal LC 10 concentration of ME-SeNPs and a subsequent 24-h recovery period, a significant and pronounced elevation in NO levels was observed, reaching 29.00 ± 3.28 nmol/L (One-way ANOVA, exact *p* < 0.05). This nitrosative challenge was further aggravated at the higher sublethal LC 25 threshold, where NO production escalated significantly to 34.61 ± 6.12 nmol/L (Table 10).

**Table 9:** Effect of Selenium Nanoparticles (ME-SeNPs) on nitric oxide (NO) concentration in *Biomphalaria alexandrina* snails.

Treatments groups	Nitric oxide concentration nmol/L
*Biomphalaria alexandrina* (Control groups)	17.29 ± 0.96
*Biomphalaria alexandrina* treated with LC10 (mg/L) of (ME-SeNPs) for 24 h then recovered for 24 h	29.00 ± 3.28
*Biomphalaria alexandrina* treated with LC_25_(mg/L) of (ME-SeNPs) for 24 h then recovered for 24 h	34.61 ± 6.12

Data are expressed as mean ± SD (n = 3 independent replicates per group). After verifying data normality and homogeneity of variance, statistical significance was determined using One-way ANOVA. Differences were considered statistically significant at *p* < 0.05 compared with the control group.

**Table 10:** Impact of Selenium Nanoparticles (ME-SeNPs) on DNA Integrity in *Biomphalaria alexandrina* snails.

Treatments	% tailed	Tail length(um)	% DNA in tail	Tail moment	Olive tail moment
*Biomphalaria alexandrina* (Control groups)	7.00 ± 0.46	11.25 ± 2.42	9.54 ± 0.41	1.25 ± 0.26	1.69 ± 0.12
*Biomphalaria alexandrina* treated with LC_10_ (mg/L) of (ME-SeNPs) for 24 h then recovered for 24 h	12.96 ± 0.46	9.74 ± 1.78	12.63 ± 1.70	1.86 ± 0.32	4.18 ± 1.78
*Biomphalaria alexandrina* treated with LC_25_ (mg/L) of (ME-SeNPs) for 24 h then recovered for 24 h	16.56 ± 0.47	11.405 ± 0.73	9.83 ± 4.36	2.59 ± 0.34	2.60 ± 1.34

Data are expressed as mean ± SD (n = 3 independent replicates per group). After verifying data normality and homogeneity of variance, statistical significance was determined using One-way ANOVA. Differences were considered statistically significant at *p* < 0.05 compared with the control group.

### Impact of selenium nanoparticles (ME-SeNPs) on DNA integrity in *Biomphalaria alexandrina* snails

**Fig. 14:**
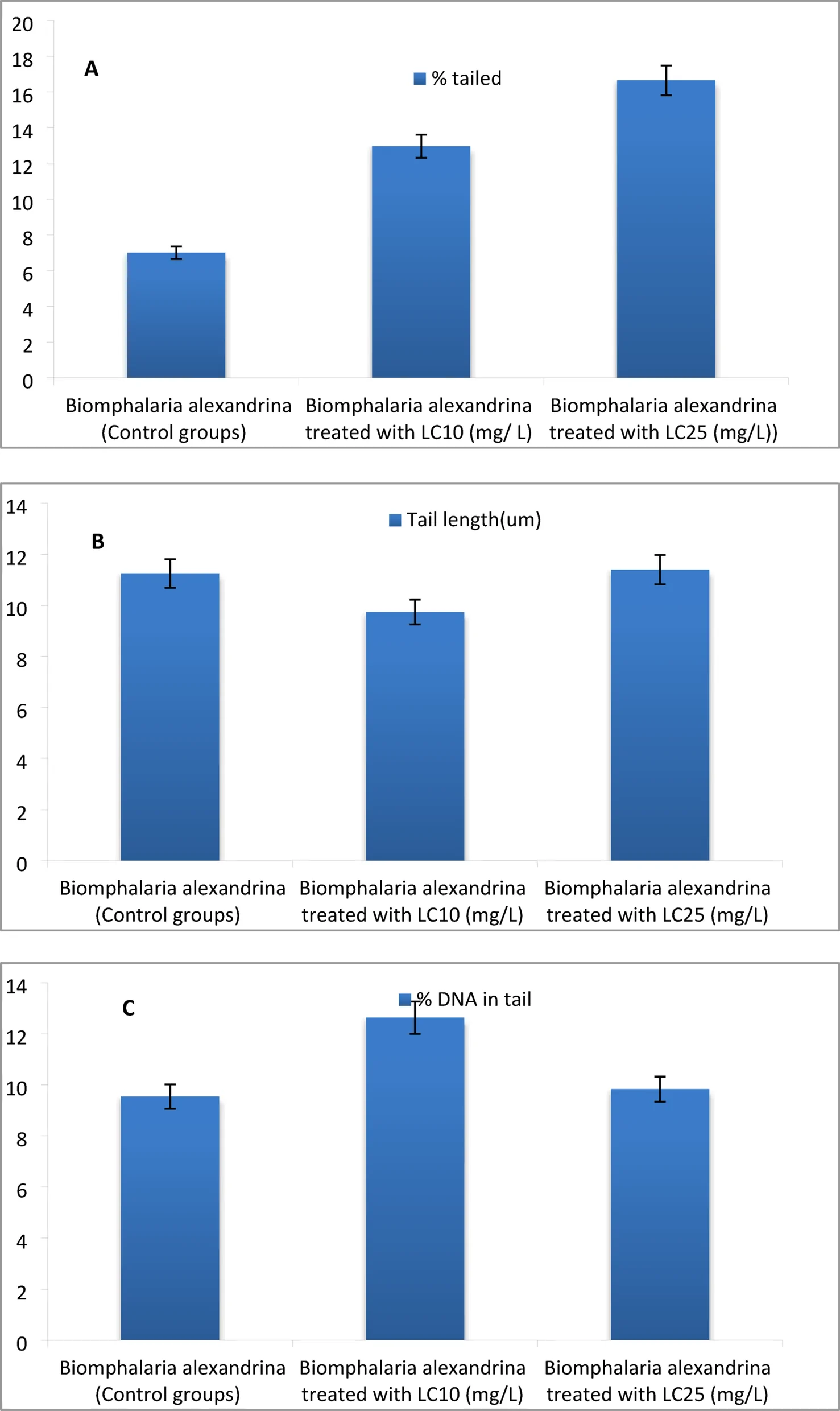

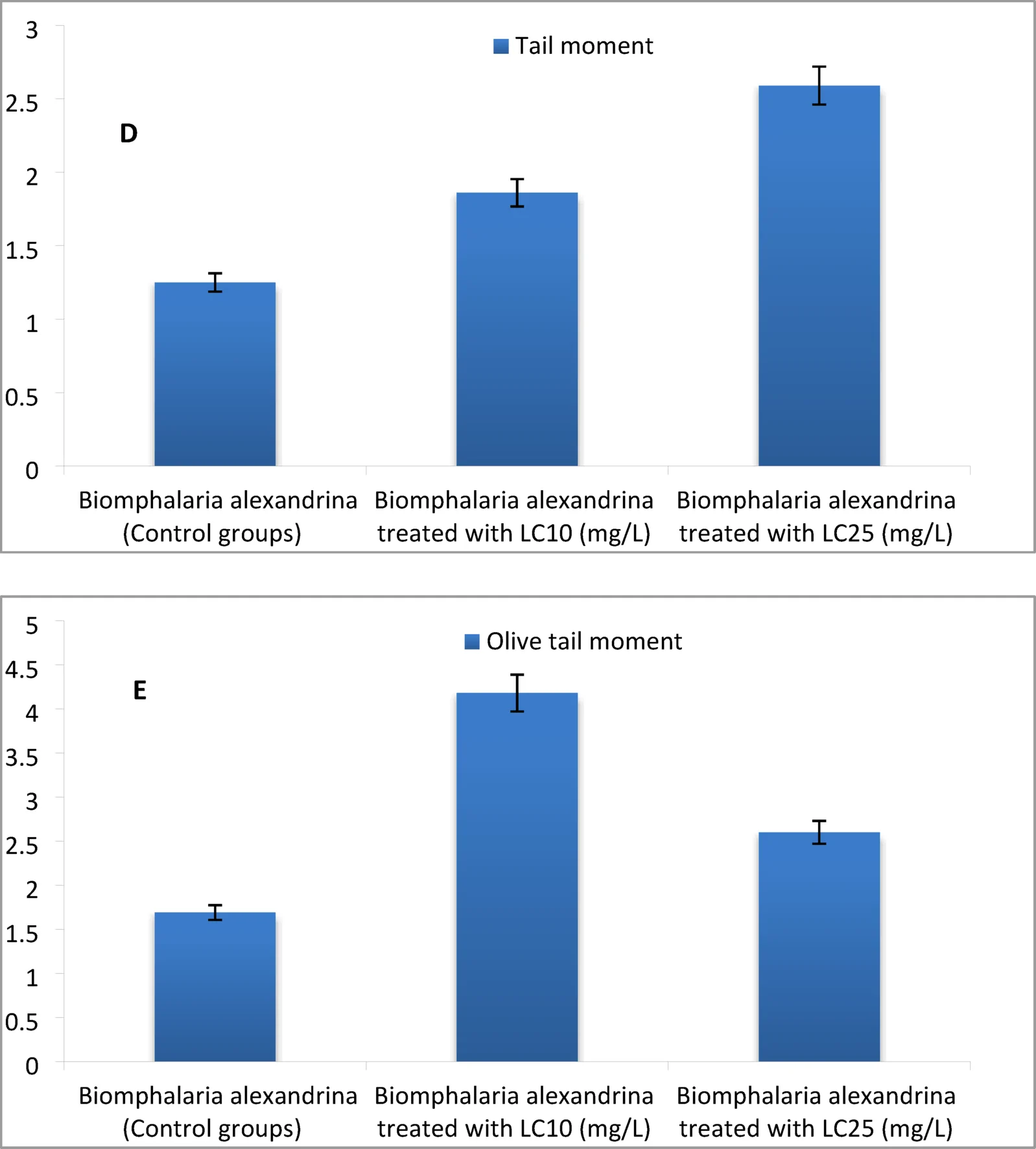
Comet assay analysis showing DNA damage in *Biomphalaria alexandrina* snails following exposure to Selenium Nanoparticles (ME*-*SeNPs). Panels (**A**–**E**) represent the percentage of tailed nuclei, tail length (µm), percentage of DNA in the tail, tail moment, and olive tail moment, respectively. Data are expressed as mean ± SD, and statistically significant differences compared with the control group are indicated at *p* < 0.05.

**Fig. 15:**
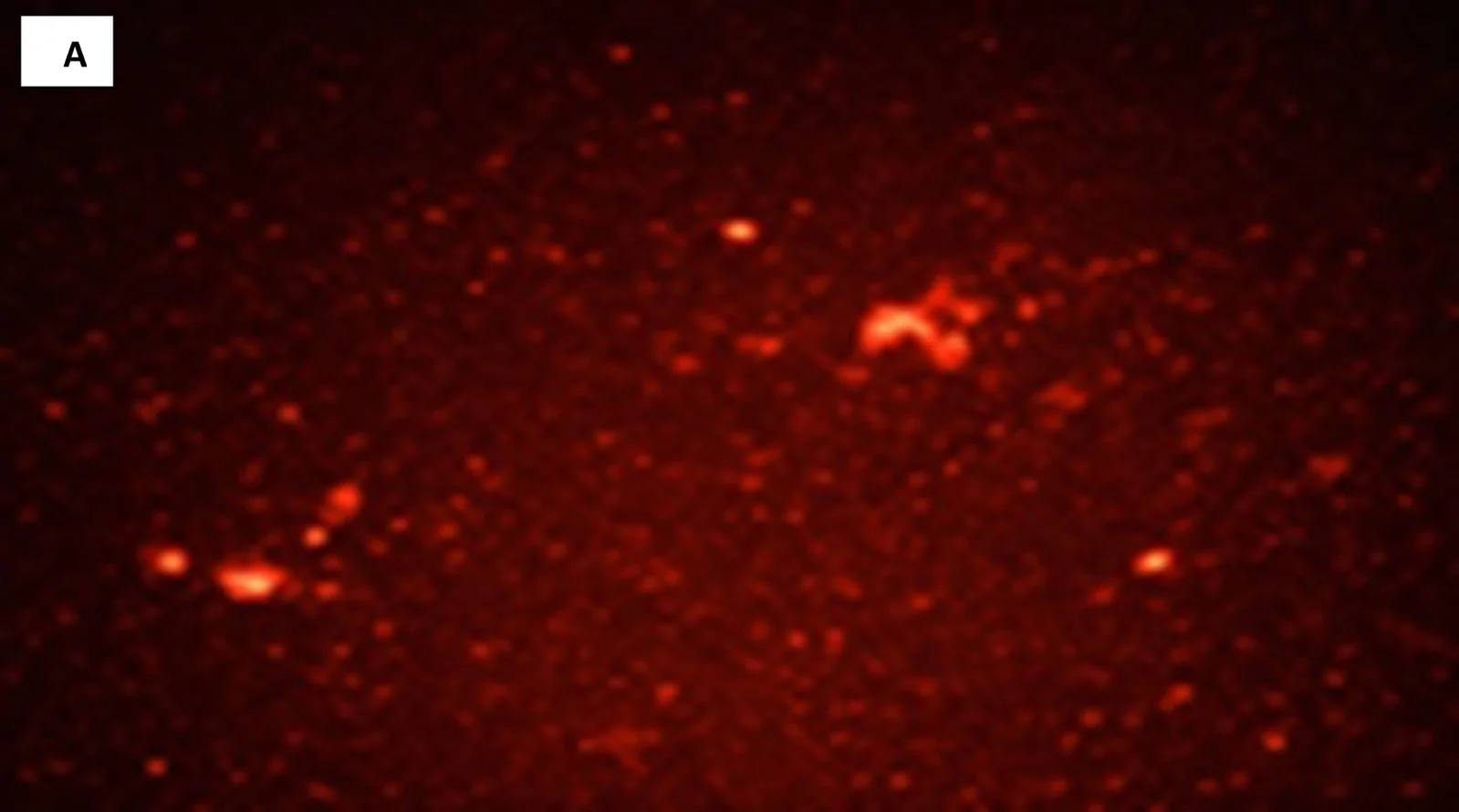

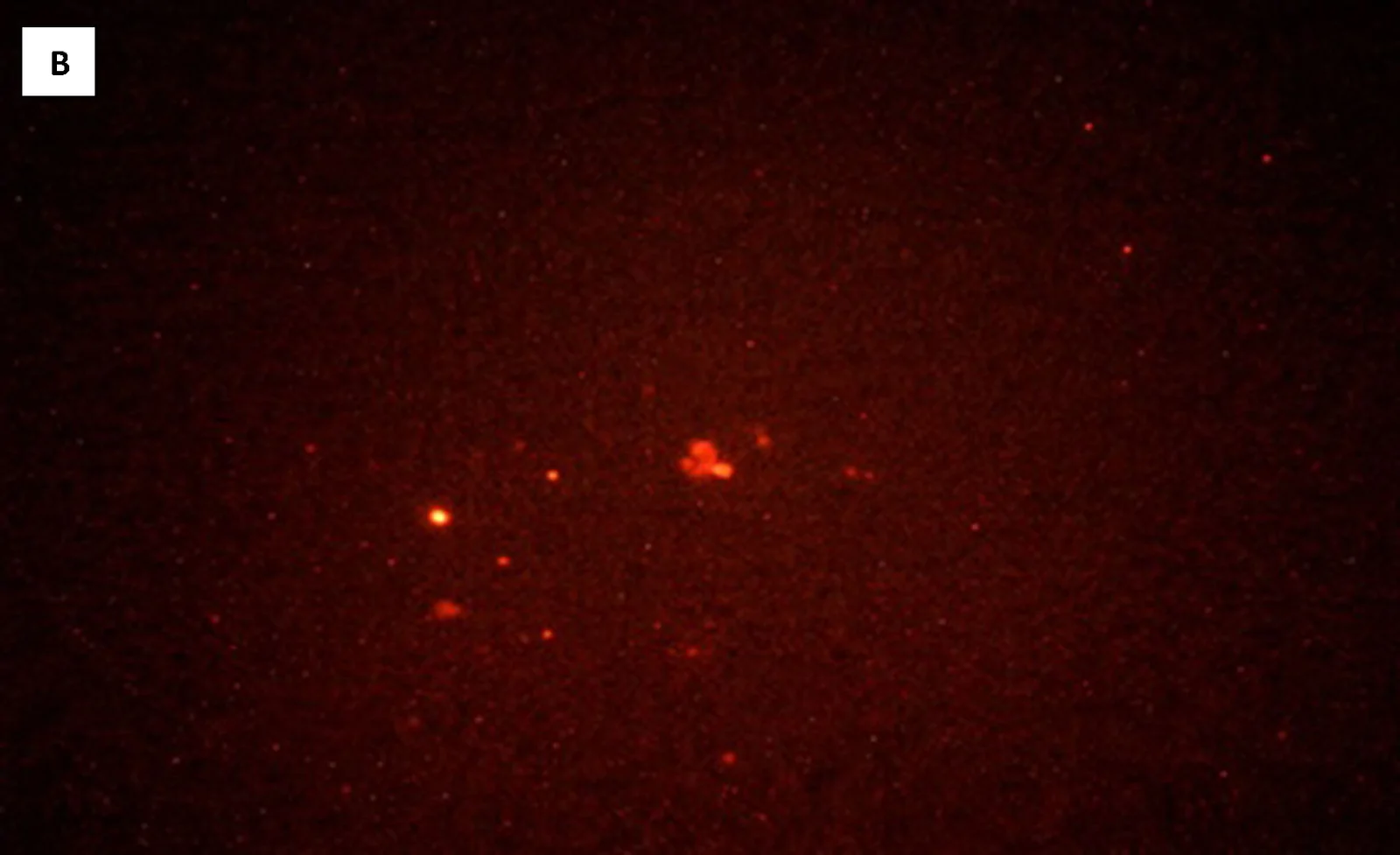

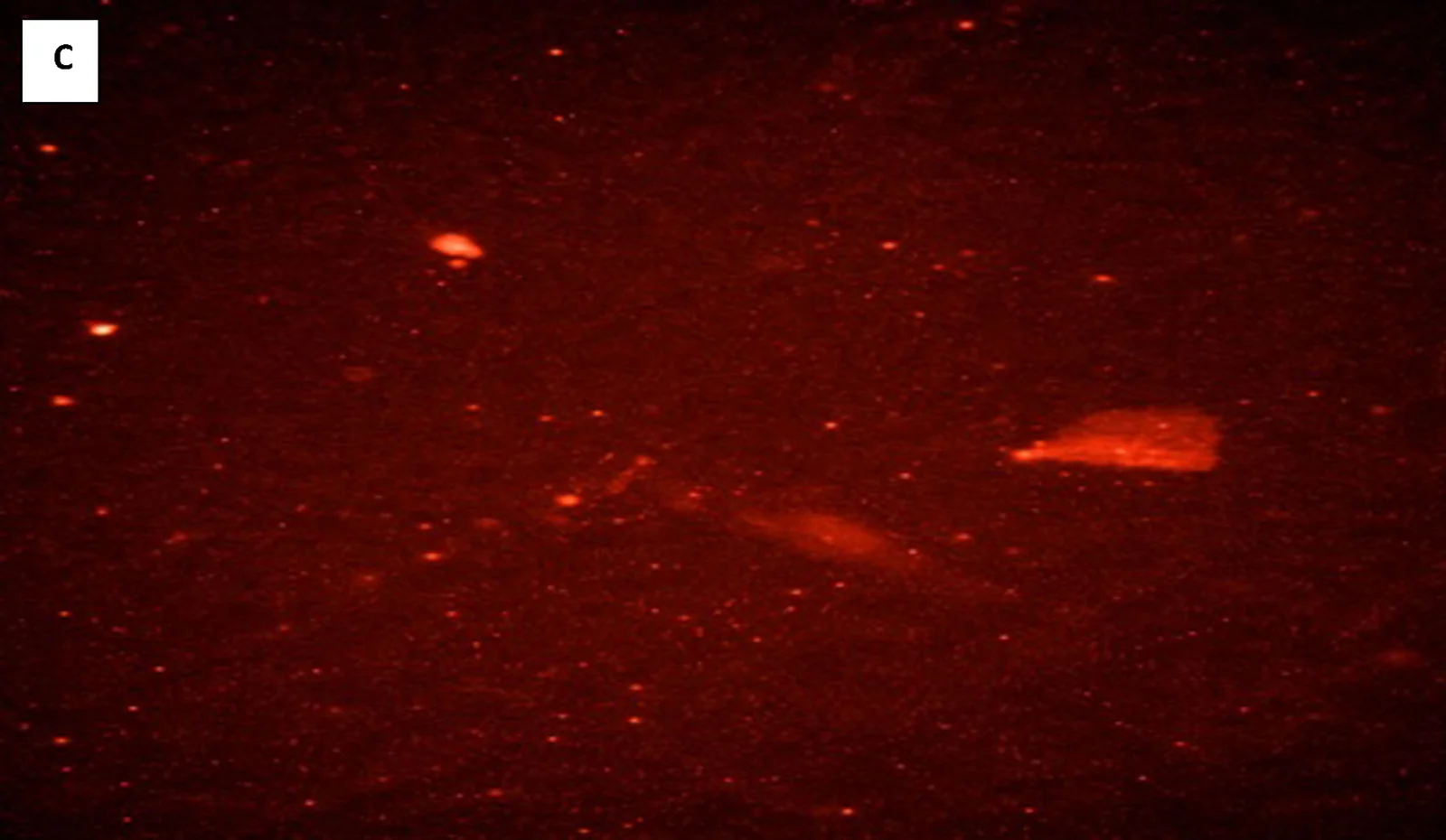
Representative comet assay images illustrating DNA damage in *Biomphalaria alexandrina* snails following exposure to Selenium Nanoparticles (ME*-*SeNPs): (**A**) control, (**B**) LC₁₀ (mg/L), and (**C**) LC₂₅ (mg/L).

Genotoxic damage in *Biomphalaria alexandrina* snails was evaluated using the comet assay to assess DNA strand breakage in the isolated whole soft tissues after shell removal (n = 3 independent replicates per group; data expressed as mean ± SD). As summarized in Table [10, Fig. 14A–D) and Fig. 15A–C], exposure to ME -SeNPs induced a concentration-dependent increase in genotoxic parameters. In the control group, baseline levels were recorded as 7.00 ± 0.462% for % tailed, 11.25 ± 2.425 µm for tail length, 9.55 ± 0.413% for DNA in the tail, and 1.26 ± 0.266 and 1.70 ± 0.124 for tail moment and Olive tail moment, respectively. At the sublethal LC 10 concentration, a statistically significant induction of DNA damage was evident, characterized by an increase in % tailed nuclei (12.97 ± 0.465%, *p* < 0.05), DNA in the tail (12.64 ± 1.704%, *p* < 0.05), and tail moment (1.87 ± 0.321, *p* < 0.05). While tail length remained stable at this concentration, the Olive tail moment showed a pronounced elevation (4.18 ± 1.786, *p* < 0.05). Upon exposure to the higher LC 25 threshold, genotoxicity was further exacerbated; the proportion of % tailed nuclei surged to 16.57 ± 0.474% (*p* < 0.05), tail length increased to 11.41 ± 0.736 µm (*p* < 0.05), and tail moment reached 2.59 ± 0.342 (*p* < 0.05).

## Discussion

Fueled by the urgent demand for environmental sustainability, green nanotechnology has advanced rapidly, accelerating the development of eco-compatible synthesis routes. Within this context, biogenic selenium nanoparticles (ME-SeNPs) have emerged as highly attractive, multifunctional candidates, largely owing to their favorable safety profiles and superior biocompatibility compared with their chemically synthesized counterparts^67,68^. A key advantage likely arises from the *Mytilus edulis* derived capping layer, which is enriched with proteins, peptides, and phenolic constituents that adsorb onto the nanoparticle surface during the reduction process. This biomolecular coating limits nanoparticle agglomeration, enhances colloidal stability, and facilitates more effective tissue interactions, thereby reducing non-specific ecotoxic effects. Accordingly, the green synthesis of selenium nanoparticles using *M. edulis* extract provides a sustainable strategy in which natural biomolecules serve as both reducing and capping agents. Successful nanoparticle formation is visually evidenced by the characteristic red orange color transition, consistent with previous reports^69^. More generally, metal nanoparticles (MNPs) including AgNPs, AuNPs, Fe₃O₄, Pt, ZnO, SeNPs, and GdNPs have received widespread attention because their nanoscale physicochemical properties translate into enhanced biological activity, particularly in antimicrobial and molluscicidal applications^28,70,71–73^. GC–MS analysis of the ethanolic extract of *Mytilus edulis* revealed a chemically diverse profile comprising lipids, carbohydrates, sterols, and aromatic compounds. The presence of key bioactive constituents such as eicosapentaenoic acid and cholesterol highlights its nutritional and therapeutic significance. These findings are consistent with Chakraborty et al. who reported that marine molluscs represent a largely underexplored source of structurally diverse bioactive compounds with promising applications in functional foods, nutraceuticals, and drug development, particularly due to their anti-inflammatory, anticancer, and immunomodulatory activities.^74^ In line with these observations, Tan et al. demonstrated that several bivalve species possess measurable total antioxidant capacity (TAC), confirming their role as natural sources of antioxidants.^75^ In the present study, HPLC analysis identified important phenolic compounds, namely chlorogenic acid and gallic acid, in *Mytilus edulis*. These polyphenols are of particular importance due to their strong antioxidant capacity, enabling them to scavenge reactive oxygen species (ROS) and reduce oxidative stress, a major contributor to chronic diseases. Chlorogenic acid plays a significant role in glucose metabolism regulation and may reduce the risk of metabolic and cardiovascular disorders, while gallic acid exhibits broad-spectrum biological activities, including anti-inflammatory, antimicrobial, and anticancer effects^76–79^.

Moreover, the redox properties of these phenolic compounds directly facilitate nanoparticle synthesis and stabilization. Supporting this mechanism, Zhu et al. demonstrated that chlorogenic and gallic acids act as natural cross-linking agents that enhance protein–polyphenol interactions, effectively improving the structural integrity and stability of such biomolecular systems^80^. This effectively aligns with findings by Mun et al., who noted that 80% ethanol provides optimal extraction efficiency and radical scavenging capacity for bioactive phenolics^81^. Despite their promising potential, marine derived phenolic compounds remain less explored than their terrestrial counterparts due to their structural complexity and the requirement for advanced analytical techniques. Nevertheless, their broad spectrum bioactivities highlight significant potential across the food, nutraceutical, and pharmaceutical sectors. In particular, these compounds offer promising therapeutic avenues for modulating gut microbiota and managing prevalent lifestyle-related disorders, including metabolic syndrome, type 2 diabetes, obesity, cancer, and Alzheimer’s disease^82^. Accordingly, characterization analyses confirmed the successful green biosynthesis of SeNPs using *Mytilus edulis* extract. UV–Visible spectroscopy revealed a characteristic absorption band at 280 nm, corresponding to the π → π* transitions of aromatic biomolecules in the capping layer and Se 4p valence band transitions, which was accompanied by a visible color change, confirming nanoparticle formation^61^. DLS analysis demonstrated a moderate particle size distribution. The negative zeta potential (− 33.13 mV) of ME-SeNPs indicates adequate electrostatic stabilization of the colloidal suspension. The biosynthesized selenium nanoparticles (ME-SeNPs) exhibit a moderate polydispersity index (PDI = 0.47), indicating size heterogeneity characterized by the co-existence of individually dispersed nanoparticles and loosely associated nanoclusters. This moderate colloidal stability is an inherent limitation of the green synthesis approach, arising from the complexity of the *Mytilus edulis*-derived biogenic capping layer. Because this size variation can introduce fluctuations in biological performance (such as cellular uptake and surface reactivity), Moreover, TEM and SEM analyses revealed well-dispersed, spherical nanoparticles with sizes ranging from 35–65 nm, a morphology that enhances biological interactions and cellular uptake^83,84^.

The larger hydrodynamic diameter of ME-SeNPs measured by DLS (~ 292 nm) compared with the TEM core size (35–65 nm) does not reflect the true inorganic core dimensions. FTIR confirms that extract-derived functional groups (hydroxyl, amine, and carboxyl) act as both reducing and capping agents, forming a cooperative multi-layer biogenic corona that provides chemical anchoring plus steric/electrostatic stabilization. Protein–SeNP interactions are supported by Amide I (1633 → 1639 cm^−1^) and Amide II (~ 1540 → 1523 cm^−1^) shifts, together with covalent anchoring through oxidized tyrosine residues. Polysaccharide stabilization is further indicated by multipoint C–OH coordination (≈1101 → 1045 cm^−1^) and stabilization of glycosidic/uronic-acid carboxylate moieties. Additionally, because DLS is an intensity-weighted technique, it is highly sensitive to larger structures, meaning the ~ 292 nm peak likely captures these reversible clusters and hydrated layers rather than isolated cores (69–71). This pronounced DLS–TEM size discrepancy is widely documented for other nanomaterials, including gold (AuNPs), iron oxide (Fe_3_ O_4_ NPs), and polymeric nanoparticles, where primary TEM sizes of 10–50 nm routinely expand to DLS diameters of 150–300 nm due to similar hydration and aggregation effects^85–88^. In biomedical applications, this DLS-derived hydrodynamic size is highly critical and physiologically relevant, as it dictates how the particles actually behave and interact within biological systems. Ultimately, TEM and DLS serve as complementary tools; the former captures the definitive size of primary cores, while the latter describes their real-world dynamics and aggregation states in solution. Importantly, the cooperative multi-layer biogenic corona and favorable surface charge of ME-SeNPs support colloidal stability and are consistent with recent advances in green nanotechnology, where selenium nanoparticle performance in antimicrobial activity and bioefficacy is strongly linked to surface chemistry and particle-size/colloidal behavior^89,90^.

The experimental antimicrobial evaluation demonstrated that ME-SeNPs exhibit clear inhibitory activity against both *Staphylococcus aureus* and *Escherichia coli*, yielding zones of inhibition measuring 13 ± 0.43 mm and 15 ± 0.58 mm, respectively. However, under the tested conditions, this antibacterial potency remained lower than that of the positive control, cefotaxime (> 22 ± 0.30 mm), which demonstrated significantly higher potency and lower MIC values. Notably, the quantified MIC value of 31.25 µg/Ml against *E. coli* highlights a pronounced efficiency over several previously reported plant-derived counterparts, positioning these *M. edulis*-stabilized nanostructures as highly reactive candidates. Time-kill kinetics further verified that ME-SeNPs exert a clear, concentration-dependent bactericidal effect against *E. coli* (evaluated at (0.5 × 1 and 2 × MIC),). Conversely, the kinetic profile against *S. aureus* indicated a predominantly bacteriostatic action. This differential susceptibility is experimentally linked to a significant contrast in antibacterial efficacy between the Gram-negative and Gram-positive strains, with *E. coli* displaying higher vulnerability. Crucially, strict control protocols including rigorous washing and centrifugation effectively eliminated free precursors from the suspension. This confirms that the observed biological potency originates solely from the engineered ME-SeNPs rather than unreacted chemical components. Beyond the observed growth inhibition and kinetic profiles, the underlying mode of action driving nanoparticle internalization and lethality is hypothesized to rely on cellular surface interactions and intracellular damage (Fig. 8). The observed higher susceptibility of *E. coli* compared to *S. aureus* is plausibly attributed to fundamental structural differences in their cell walls. The thick peptidoglycan layer of *S. aureus* (20–8 nm) likely acts as a robust physical barrier that restricts nanoparticle penetration, whereas the significantly thinner peptidoglycan layer of *E. coli* (2–3 nm) may facilitate easier membrane disruption and subsequent internalization^91,92,^. Literature confirms that SeNP efficacy varies widely across species depending on synthesis methods, surface chemistry, and stabilizing biomolecules^28,93,94^. For instance, particle size and capping matrices critically dictate bioactivity, as seen in orange peel waste-derived SeNPs (18.3 nm) yielding a 35 mm inhibition zone against MRSA at 25 µg/mL^95^, and ultra-small SeNPs (8.08 nm) targeting *Fusarium oxysporum* at 250 µg/mL^96^. Similarly, SeNPs synthesized via *Ferula assa-foetida* (90.4 nm) and *Fusarium fujikuroi* (10–19 nm) induced substantial inhibition zones against Gram-negative *E. coli* ^40,97^, whereas a mycosynthesized formulation reached a low MIC of 9.37 µg/mL against *S. aureus*^91^. Supporting the importance of coatings, Lyu et al. demonstrated that EGCG-functionalized SeNPs exhibited strong antimicrobial activity in food systems with proven in vivo biosafety in zebrafish^27^.

In our proposed model, the differential strain susceptibility is strongly influenced by a complex interplay between the negative surface charge (− 33.13 mV) of ME-SeNPs and the functionalized *M. edulis* capping layer, which is highly enriched in proteins, phenolic compounds, chitin biomolecules, and marine fatty acids^98,99^. This biogenic corona is hypothesized to actively chelate stabilizing divalent cations (Mg^2+^ and Ca^2+^) within the lipopolysaccharide (LPS) layer of *E. coli*. This chelation process potentially destabilizes the outer membrane, thereby promoting nanoparticle internalization. Once inside the cell, the nanoparticles are proposed to induce severe intracellular oxidative stress and subsequent DNA damage^98,99^. This configuration, supported by UV–Vis and TEM analysis (35–65 nm core), is also thought to facilitate tissue uptake in *Biomphalaria alexandrina*, driving its dose-dependent molluscicidal efficacy^61^. While metallic nanostructures like silver or Ag–Se bimetallic nanoparticles often exhibit superior activity due to enhanced oxidative stress^100^, biogenic designs offer a highly promising alternative with remarkable biosafety profiles. For example, Mercado et al. isolated potent, non-cytotoxic antimicrobial peptides (AMPs) from Mytilus edulis chilensis gills with broad-spectrum efficacy^101^. Furthermore, literature indicates that BSA-stabilized SeNPs (20–60 nm) exhibit a seven-fold reduction in acute toxicity (LD 50 = 113 mg Se/kg) in mice compared to sodium selenite (LD 50 = 15 mg Se/kg), while maintaining equivalent efficacy in upregulating critical antioxidant selenoenzymes^102,104^.

The biosynthesized *Mytilus edulis*-derived selenium nanoparticles (ME-SeNPs) exhibited pronounced, dose-dependent toxicity against *Biomphalaria alexandrina* (shell diameter: 8–10 mm), yielding specific lethal concentrations of LC 10 (86.89 mg/L), LC 25 (110.06 mg/L), LC 50 (135.72 mg/L), and LC 90 (184.48 mg/L). These findings strongly align with Morad et al., who reported significant molluscicidal and larvicidal potential of green-synthesized SeNPs against *B. alexandrina* and *Schistosoma mansoni*^104^. This potent efficacy is driven by a multi-targeted physiological disruption orchestrated by the specific chemical fingerprint of the *M. edulis* capping layer. HPLC profiling revealed a dominant phenolic framework of Gallic acid (61.25%) and Chlorogenic acid (38.74%) functioning as key stabilizing agents^72,98^, coupled with marine-derived antimicrobial peptides (AMPs) possessing cationic and amphipathic structures^105–107^. This biochemical combination significantly enhances the bioavailability, tissue dispersion, and membrane permeability of the nanoplatform, facilitating its deep internalization within the host snails.

Compared to established chemical molluscicides, such as the advanced niclosamide formulations reported by Jian-rong et al. which achieve complete control at remarkably low concentrations (e.g., 0.125 mg/L), the green-synthesized ME-SeNPs exhibited a higher LC 50 value of 135.72 mg/L (95% CL: 96.10–161.86 mg/L)^108^. From an ecological perspective, however, the primary merit of biogenic ME-SeNPs lies in their biocompatible profile and potential for biodegradable environmental clearance rather than immediate dose-potency. Nevertheless, while the *M. edulis*-mediated green synthesis minimizes initial chemical toxicity, the expanding environmental discharge of nanomaterials raises valid ecotoxicological concerns regarding bioaccumulation and long-term vulnerabilities in living systems^109–112^.

To contextualize the systemic impact of these nanoparticles, it is essential to understand the innate immune architecture of the Phylum Mollusca. Encompassing diverse aquatic and terrestrial taxa such as bivalves, cephalopods, and gastropods, molluscan immunity relies fundamentally on hemocytes as the pivotal cellular lineage mediating defense through phagocytosis-driven respiratory bursts and antimicrobial secretion. ^113^ Morphologically categorized into hyaline, semigranular, and granular cells, the abundance and functional roles of these hemocyte subtypes vary significantly across different molluscan taxa, driven by endogenous physiology and environmental exposure to pathogens like protozoans, bacteria, and viruses. ^114^When foreign agents encounter host cells, cell-surface or soluble Pattern Recognition Receptors (PRRs), such as complement factors, sense the threat to initiate defensive reactions, monitoring both microbial patterns and environmental shifts to maintain organismal balance^115^.

To interact with these immune cells in vivo, engineered nanomaterials (ENMs) must breach physical anatomical barriers, where invertebrates rely on protective cuticles or peritrophic membranes rather than the mucosal traps deployed by vertebrates^116,117^. Phagocytosis occurs only if ENMs breach these barriers, where phagocytes either destroy engulfed particles or permanently sequester indigestible materials to preserve tissue integrity. However, establishing a predictive correlation between ENM properties (size, shape, stiffness, surface charge) and cellular uptake remains elusive, leaving safe-by-design nanotechnology unfulfilled since host physiology ultimately dictates the outcome^117,118^. To date, most fundamental data on bivalve hemocyte–ENM interactions have been obtained from in vitro models. Research on *Mytilus galloprovincialis* showed that nano-carbon black (NCB) causes concentration-dependent uptake, rapid extracellular reactive oxygen species (ROS), lysozyme, and nitric oxide (NO) release, alongside pre-apoptotic processes mediated by mitogen-activated protein kinase (MAPK) activation. ^119^ This confirms that ENMs induce mammalian-like inflammatory responses in mussels, proving bivalve immunocytes are excellent models for marine invertebrate ENM research, with similar immunotoxic and immunomodulatory effects observed across various bivalves like clams and oysters depending on particle type and exposure settings^120^.

In *M. galloprovincialis*, the interaction between hemolymph serum (HS) and nanoparticles dictates immunotoxic outcomes through selective biomolecular corona formation^121^. For instance, 50 nm positively charged amino-modified polystyrene (PS-NH_2_) selectively binds the serum opsonin MgC1q6^122^, accelerating cellular damage and ROS production via p38 MAPK dysregulation^123^, while in vivo repeated exposure upregulates MgC1q6 expression and boosts bactericidal activity^124^. Conversely, larger 100 nm PS-NH_2_ particles display an absence of agglomeration and inversion of the zeta potential, showing reduced lysosomal toxicity and weaker effects on phagocytosis by binding a different complement component, MgC1q44^125^. Similarly, decreased ROS production by hemocytes exposed to cerium nano-oxide (n-CeO_2_) was associated with a biocorona involving Cu, Zn-superoxide dismutase (SOD). This indicates that biocorona formation is ENM-selective and utilizes distinct soluble serum factors acting as PRRs to mediate immune interactions and determine functional outcomes^121^. When shifting focus to gastropod models, sublethal exposure to biogenic ME-SeNPs at LC 10 (86.89 mg/L) and LC 25 (110.06 mg/L) elicits a striking biological paradox in *B. alexandrina* snails (*P* < 0.05). Upon internalization of these sublethal nanomaterials, an immediate intracellular accumulation of ROS occurs alongside a marked, dose-dependent surge in intracellular NO production relative to the untreated control (*P* < 0.05). This signals the concurrent activation of oxidative and nitrosative stress cascades^126,127^. This early-stage oxidative and nitrosative challenge prompts an immediate compensatory immune response characterized by marked hemocyte proliferation as a core defense mechanism to alter hemolymph composition for contaminant transport and antioxidant deployment. This process potentially modulates key upstream cellular regulators, such as the Keap1–Nrf_2_ signaling pathway, in an attempt to restore physiological equilibrium^104^'^128^–131. However, this initial proliferative upregulation concurrently bridges the transition toward chronic systemic toxicity rather than resolution. The resulting intracellular NO accumulation functions as a dual-acting signaling burden: it stimulates hemocyte defensive responses, but simultaneously undermines genomic integrity as the sustained generation of ROS and NO completely overwhelms the snail’s baseline antioxidant capacity. Once the pro-inflammatory microenvironment shifts from a compensatory defense to a destructive cascade, this concentration-dependent surge in intracellular NO inflicts severe genotoxicity (*P* < 0.05), breaching the nucleus and resulting in lipid peroxidation, protein degradation, and downstream genomic damage^104^.

## Strengths and limitations

### Strengths and limitations

This study highlights the bio-evaluative potential and eco-friendly synthesis of selenium nanoparticles utilizing *Mytilus edulis* extract (ME-SeNPs) as a sustainable bio-reducing and capping agent, avoiding hazardous chemicals. A major strength of this formulation lies in its dual biological efficacy, demonstrating potent antibacterial activity against both Gram-positive (*Staphylococcus aureus*) and Gram-negative (*Escherichia coli*) pathogens, alongside high molluscicidal performance against *Biomphalaria alexandrina*. Mechanistically, the molluscicidal action is comprehensively driven by induced nitrosative stress, widespread oxidative and structural DNA damage, and severe immunomodulatory disruption, underscoring its promising role as a multi-target agent for controlling the intermediate host of schistosomiasis. Despite these notable advantages, several limitations warrant consideration. First, the antibacterial evaluation was confined to planktonic bacterial strains, leaving its anti-biofilm potential unexplored under dynamic physiological conditions. Second, toxicity assessments were primarily focused on the target aquatic organisms; comprehensive in vitro cytotoxicity profiles on human cell lines as well as in vivo mammalian ecotoxicological safety studies remain essential before broader application. Lastly, the chemical stability, persistence, degradation kinetics, and overall environmental fate of ME-SeNPs under complex natural field conditions require rigorous long-term evaluation.

### Future perspectives

To build upon these foundational findings and facilitate practical translation, future investigations should employ advanced multi-omics approaches specifically transcriptomic and proteomic analyses to fully delineate the precise molecular and cellular pathways driving ME-SeNPs-induced toxicity. Further biological assays should expand to evaluate anti-biofilm efficacy against mature bacterial communities under static and microfluidic flow conditions. In parallel, thorough in vivo pharmacokinetic, toxicokinetic, and mammalian systemic safety profiling are critically needed to establish safe therapeutic margins and occupational handling parameters. Finally, scaling up to field-simulated mesocosm trials will be imperative to monitor the long-term environmental behavior, bioaccumulation potential, degradation dynamics, and eco-safety profiles of these biogenic nanoparticles within realistic aquatic ecosystems.

### Alignment with SDGs (sustainable development goals)

ME-SeNPs target *S. aureus* ATCC 25923, *E. coli* ATCC 25922, and intermediate hosts to suppress schistosomiasis (SDG 3), providing a green alternative to toxic synthetic molluscicides (SDGs 6 & 14).

## Conclusion

This study successfully demonstrates the green synthesis of stable selenium nanoparticles (SeNPs) utilizing *Mytilus edulis* extract. The synthesized nanoparticles exhibited potent antimicrobial activity against *Staphylococcus aureus ATCC 25923 and Escherichia coli ATCC 25922* and significant molluscicidal activity against *Biomphalaria alexandrina* snails.

## Supplementary Information

Below is the link to the electronic supplementary material.

Supplementary Material 1

Supplementary Material 2

Supplementary Material 3

Supplementary Material 4

## Data Availability

In this study, we obtained the reference strains Escherichia coli ATCC 25922 was from the ATCC collection https://www.atcc.org/products/25922 Staphylococcus aureus ATCC 25923 was from the ATCC collection https://www.atcc.org/products/25923. Cefotaxime was purchased from Sigma-Aldrich and used for antibiotic susceptibility testing. Chemical structure and properties are available at https://pubchem.ncbi.nlm.nih.gov/compound/5742673
