# Supplementary material for: Mytilus edulis-mediated green synthesis of selenium nanoparticles with antimicrobial and molluscicidal applications
Source: Sci Rep. 2026 Sep 7;16:27918. doi: 10.1038/s41598-026-65860-3 (PMC13550546; doi:10.1038/s41598-026-65860-3)
Supplement: Supplementary file 1 [file 41598_2026_65860_MOESM1_ESM.pdf]

Data File C:\CHEM32\1\DATA\PP 29-9-2025 2025-09-29 04-17-29\006-0601.D
Sample Name: dr salwa 1

===

Acq. Operator : SYSTEM   Seq. Line : 6
Acq. Instrument : hplc -2   Location : Vial 6
Injection Date : 9/29/2025 6:52:17 AM   Inj : 1
Inj Volume : 5.000 µl
Acq. Method : C:\CHEM32\1\DATA\PP 29-9-2025 2025-09-29 04-17-29\POLYPHENOL 2023.M
Last changed : 9/29/2025 4:17:29 AM by SYSTEM
Analysis Method : C:\CHEM32\1\DATA\PP 29-9-2025 2025-09-29 04-17-29\POLYPHENOL 2023.M (Sequence Method)
Last changed : 10/13/2025 11:54:18 PM by SYSTEM
(modified after loading)
Additional Info : Peak(s) manually integrated

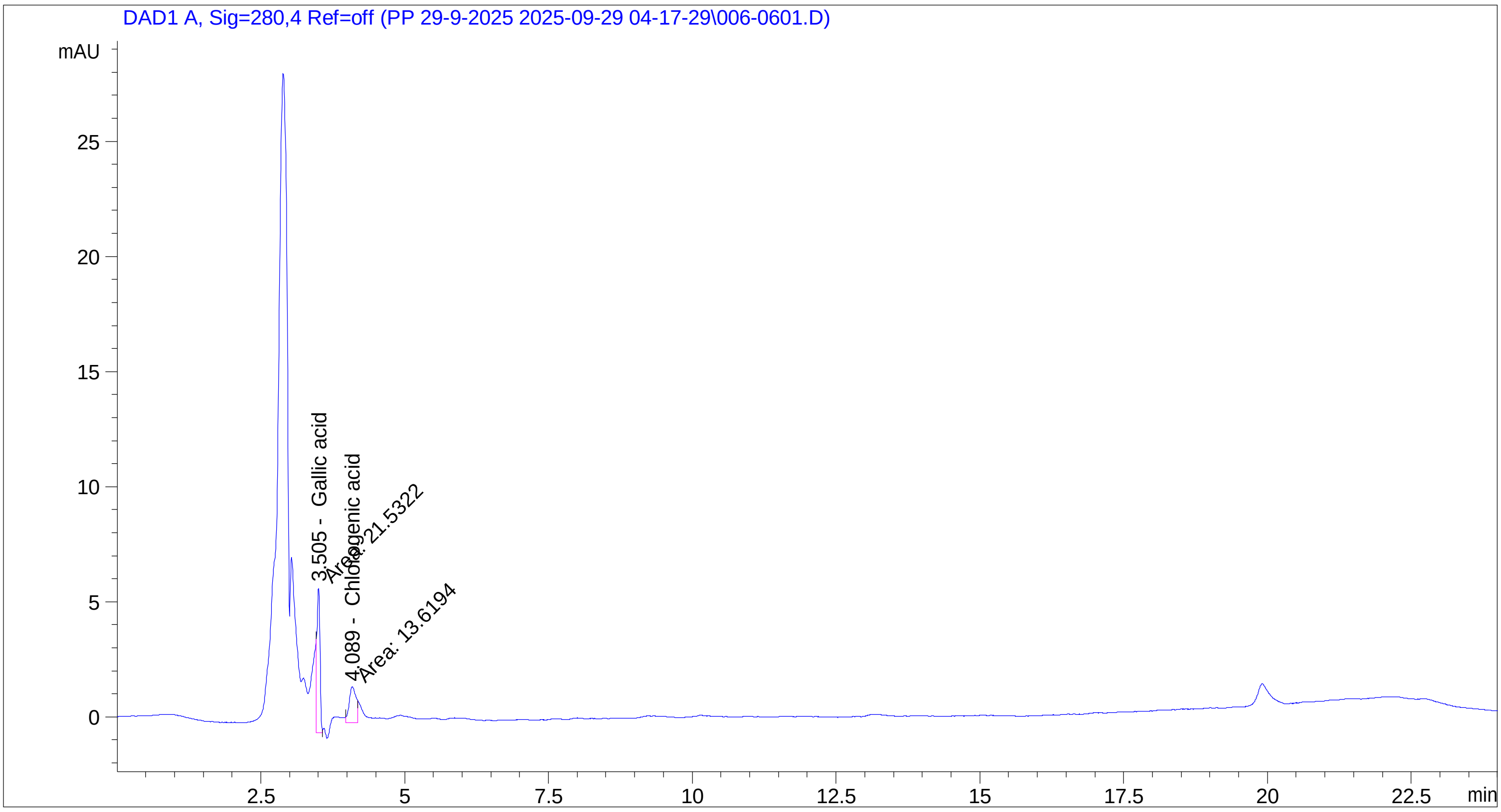

===

Area Percent Report

===

Sorted By : Signal
Calib. Data Modified : 9/29/2025 9:11:50 AM
Multiplier : 1.0000
Dilution : 1.0000
Do not use Multiplier & Dilution Factor with ISTDs

Signal 1: DAD1 A, Sig=280,4 Ref=off

Peak #	RetTime [min]	Type	Width [min]	Area [mAU*s]	Area %	Name
1	3.505	MM	0.0570	21.53222	61.2553	Gallic acid
2	4.089	MM	0.1443	13.61941	38.7447	Chlorogenic acid
3	4.423		0.0000	0.00000	0.0000	Catechin
4	5.399		0.0000	0.00000	0.0000	Methyl gallate
5	5.821		0.0000	0.00000	0.0000	Coffeic acid

Peak #	RetTime [min]	Type	Width [min]	Area [mAU*s]	Area %	Name
6	6.306		0.0000	0.00000	0.0000	Syringic acid
7	6.892		0.0000	0.00000	0.0000	Rutin
8	7.199		0.0000	0.00000	0.0000	Ellagic acid
9	8.541		0.0000	0.00000	0.0000	Coumaric acid
10	8.920		0.0000	0.00000	0.0000	Vanillin
11	9.589		0.0000	0.00000	0.0000	Ferulic acid
12	10.364		0.0000	0.00000	0.0000	Naringenin
13	11.475		0.0000	0.00000	0.0000	Rosmarinic acid
14	15.943		0.0000	0.00000	0.0000	Daidzein
15	17.298		0.0000	0.00000	0.0000	Querectin
16	19.140		0.0000	0.00000	0.0000	Cinnamic acid
17	20.082		0.0000	0.00000	0.0000	
18	20.617		0.0000	0.00000	0.0000	Kaempferol
19	21.189		0.0000	0.00000	0.0000	Hesperetin

Totals : 35.15163

4 Warnings or Errors :

Warning : Calibration warnings (see calibration table listing)
Warning : Calibrated compound(s) not found
Warning : Invalid calibration curve, (Gallic acid)
Warning : Invalid calibration curve, (Chlorogenic acid)

*** End of Report ***
