# Supplementary material for: Mytilus edulis-mediated green synthesis of selenium nanoparticles with antimicrobial and molluscicidal applications
Source: Sci Rep. 2026 Sep 7;16:27918. doi: 10.1038/s41598-026-65860-3 (PMC13550546; doi:10.1038/s41598-026-65860-3)
Supplement: Supplementary file 2 [file 41598_2026_65860_MOESM3_ESM.pdf]

Data File C:\CHEM32\1\DATA\PP 17-4-2025 2025-03-30 02-04-09\001-0101.D
Sample Name: Polyphenols STD

===

Acq. Operator	: SYSTEM	Seq. Line :	1
Acq. Instrument	: hplc -2	Location :	Vial 1
Injection Date	: 3/30/2025 2:05:54 AM	Inj :	1
		Inj Volume :	5.000 µl

Acq. Method : C:\CHEM32\1\DATA\PP 17-4-2025 2025-03-30 02-04-09\POLYPHENOL 2023.M
Last changed : 3/30/2025 2:04:09 AM by SYSTEM
Analysis Method : C:\CHEM32\1\DATA\PP 17-4-2025 2025-03-30 02-04-09\POLYPHENOL 2023.M (Sequence Method)
Last changed : 3/31/2025 12:25:03 AM by SYSTEM
(modified after loading)
Additional Info : Peak(s) manually integrated

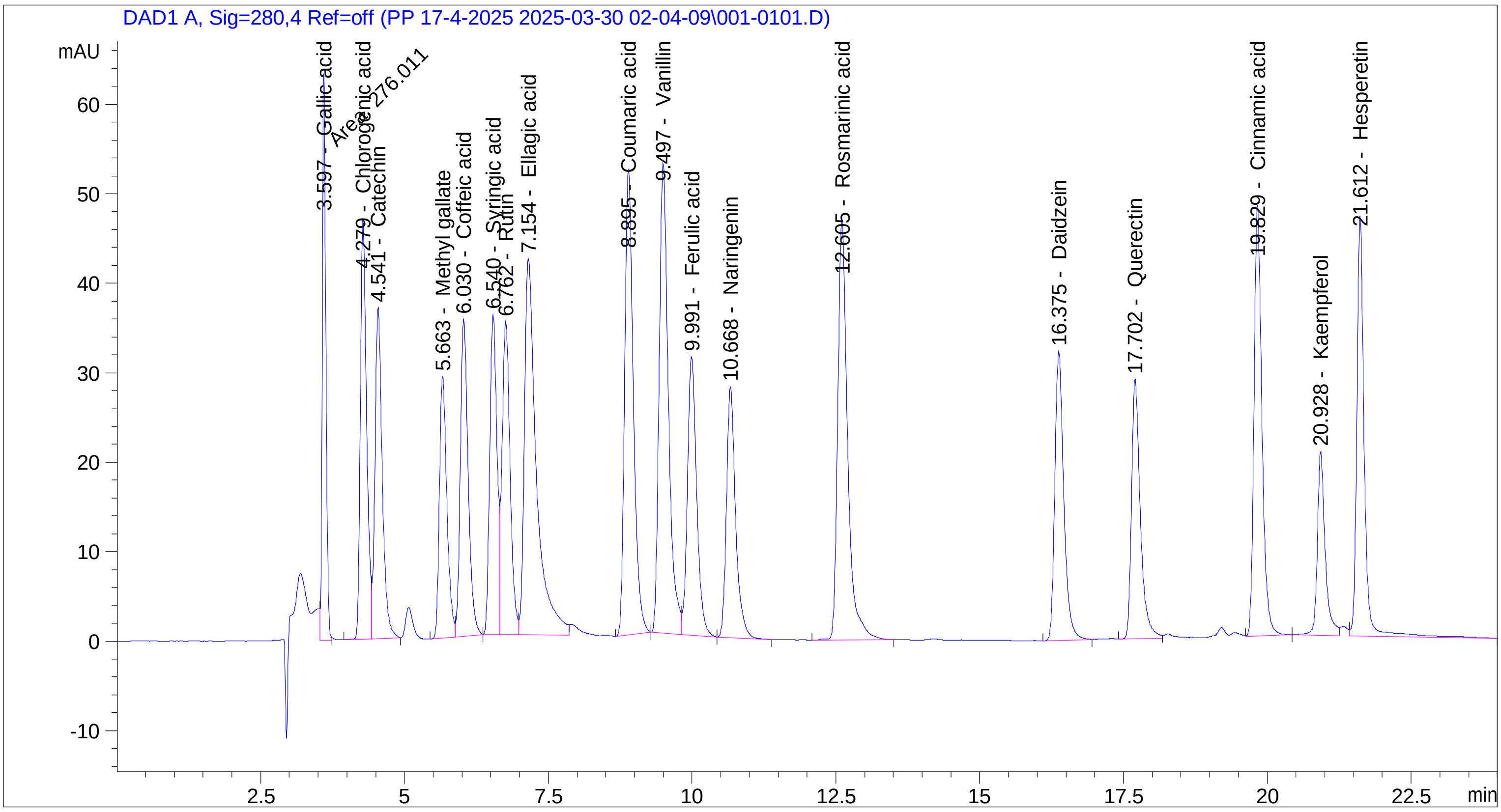

===

Area Percent Report

===

Sorted By : Signal
Calib. Data Modified : Sunday, March 30, 2025 10:15:39 PM
Multiplier : 1.0000
Dilution : 1.0000
Do not use Multiplier & Dilution Factor with ISTDs

Signal 1: DAD1 A, Sig=280,4 Ref=off

Peak #	RetTime [min]	Type	Width [min]	Area [mAU*s]	Area %	Name
1	3.597	MM	0.0724	276.01129	3.9871	Gallic acid
2	4.279	BV	0.1151	356.81793	5.1544	Chlorogenic acid
3	4.541	VB	0.1319	323.03320	4.6663	Catechin
4	5.663	BV	0.1399	264.59393	3.8221	Methyl gallate
5	6.030	VB	0.1443	334.09387	4.8261	Coffeic acid

Peak #	RetTime [min]	Type	Width [min]	Area [mAU*s]	Area %	Name
6	6.540	BV	0.1360	312.40894	4.5129	Syringic acid
7	6.762	VV	0.1469	343.90744	4.9679	Rutin
8	7.154	VV	0.2127	615.65686	8.8934	Ellagic acid
9	8.895	BB	0.1608	547.68005	7.9114	Coumaric acid
10	9.497	BV	0.1713	590.89001	8.5356	Vanillin
11	9.991	VB	0.1697	345.81433	4.9954	Ferulic acid
12	10.668	BB	0.1672	310.30411	4.4824	Naringenin
13	12.605	BB	0.1821	562.73413	8.1289	Rosmarinic acid
14	16.375	BB	0.1645	349.79211	5.0529	Daidzein
15	17.702	BV	0.1603	308.87659	4.4618	Querectin
16	19.829	VB	0.1461	462.02203	6.6741	Cinnamic acid
17	20.928	BV	0.1430	195.06477	2.8178	Kaempferol
18	21.612	VBA	0.1349	422.94934	6.1096	Hesperetin

Totals : 6922.65096

19 Warnings or Errors (10 first messages follow) :

Warning : Calibration warnings (see calibration table listing)
Warning : Invalid calibration curve, (Gallic acid)
Warning : Invalid calibration curve, (Chlorogenic acid)
Warning : Invalid calibration curve, (Catechin)
Warning : Invalid calibration curve, (Methyl gallate)
Warning : Invalid calibration curve, (Coffeic acid)
Warning : Invalid calibration curve, (Syringic acid)
Warning : Invalid calibration curve, (Rutin)
Warning : Invalid calibration curve, (Ellagic acid)
Warning : Invalid calibration curve, (Coumaric acid)

==

*** End of Report ***
